# Immune Checkpoint Inhibitors and Hypothyroidism: An Emerging Public Health Challenge Identified Through a Systematic Review of ClinicalTrials.gov

**DOI:** 10.7759/cureus.117019

**Published:** 2026-09-27

**Authors:** Jeffrey Wu, Xuetao Wu, Hong Ren

**Affiliations:** 1 Independent Research, Belmont High School, Belmont, USA; 2 Psychological and Brain Sciences, Microbiology, University of Massachusetts Amherst, Amherst, USA; 3 Internal Medicine, Virginia Mason Medical Center, Federal Way, USA

**Keywords:** hypothyroidism, immune-checkpoint inhibitors, immunotherapy, registry, surveillance

## Abstract

Immune checkpoint inhibitors (ICIs) have transformed cancer treatment, but their growing use has exposed a class of immune-related side effects with lasting consequences, most notably hypothyroidism. Unlike many other drug-related toxicities, ICI-associated hypothyroidism is frequently permanent, requiring lifelong thyroid hormone replacement. As the number of long-term cancer survivors continues to rise, this condition is an underrepresented public health problem. This study aimed to characterize how often hypothyroidism is reported as an adverse event (AE) across ICI clinical trials using publicly available data from ClinicalTrials.gov and to examine the public health implications of these findings. We expected hypothyroidism to be common, particularly with programmed cell death protein 1 (PD-1)/programmed death ligand 1 (PD-L1) inhibitors and combination regimens, and that registry-reported frequencies would be high enough to justify wider surveillance beyond individual clinical care. Using a PRISMA 2020-aligned registry-based systematic review, we screened interventional oncology trials with posted results and extracted thyroid-related AE data for descriptive analysis. Across eligible trials, hypothyroidism was consistently reported, with trial-reported proportions ranging from low single digits to over 30%, with the highest frequencies seen in combination ICI regimens. These findings demonstrate that ICI-associated hypothyroidism is frequently reported across clinical trials and support the need for standardized thyroid monitoring, long-term endocrine follow-up, and integration of thyroid toxicity surveillance into survivorship care and health system planning.

## Introduction and background

Immune checkpoint inhibitors (ICIs) are a class of cancer immunotherapies that enhance antitumor responses by blocking inhibitory pathways that normally regulate T-cell activity. ICIs have fundamentally transformed cancer therapy by restoring immune recognition and improving survival across a wide range of solid tumors and hematologic malignancies. The major classes of ICIs target key immune checkpoint pathways involved in maintaining immune tolerance, including cytotoxic T-lymphocyte-associated antigen 4 (CTLA-4), programmed cell death protein 1 (PD-1), and programmed death ligand 1 (PD-L1). Examples include the CTLA-4 inhibitor ipilimumab, PD-1 inhibitors such as nivolumab and pembrolizumab, and PD-L1 inhibitors such as atezolizumab, durvalumab, and avelumab. Blocking these pathways enhances antitumor immune responses but can also disrupt peripheral immune tolerance and cause immune-related adverse events (irAEs) [1-3].

As ICI indications expand into earlier lines of treatment and adjuvant settings, more patients are receiving these drugs and surviving long enough to experience their long-term effects [4]. That success comes with a cost because ICIs are associated with irAEs that come from loss of peripheral immune tolerance. The most common thyroid irAE is destructive thyroiditis, which typically presents as a transient thyrotoxic phase caused by the release of preformed thyroid hormone from damaged thyroid tissue, followed by hypothyroidism as hormone stores are depleted. Multiple systematic and/or narrative reviews have reported that endocrine toxicities are among the most common, and thyroid dysfunction, particularly hypothyroidism, stands out as the single most frequently reported endocrine complication associated with ICI therapy [5-7]. This is clinically significant because, unlike many other irAEs, which can be resolved after ceasing treatment, hypothyroidism often becomes permanent. Affected patients require lifelong levothyroxine replacement therapy and medical monitoring for the rest of their lives [8]. Reported rates of ICI-associated hypothyroidism vary considerably across the literature, ranging from under 5% to more than 20%, depending on the specific agent, treatment combination, and patient population studied [9-11]. While individual trials and meta-analyses have examined this issue, most are limited to specific tumor types or drug classes. ClinicalTrials.gov offers trial-level adverse-event reporting across registered studies involving a broad range of cancer types and ICI regimens. Consistent with the FDA guidance, safety data are collected throughout all phases of clinical development and represent a critical component of clinical trial evaluation. These data are commonly reported in ClinicalTrials.gov after trial completion. Although sponsors and investigators may initially record thyroid dysfunction-associated adverse events (AEs) using varying clinical descriptions, safety results are typically reported using Medical Dictionary for Regulatory Activities (MedDRA) preferred terms in ClinicalTrials.gov. The reported safety data often lack sufficient clinical detail to distinguish hypothyroidism from related thyroid toxicities, such as thyroiditis, transient thyrotoxicosis preceding hypothyroidism, or permanent hypothyroidism. Therefore, the present analysis focuses on events explicitly reported under the MedDRA preferred term hypothyroidism.

In this study, we conducted a Preferred Reporting Items for Systematic Reviews and Meta-Analyses (PRISMA) 2020-aligned systematic review of ClinicalTrials.gov-posted results to characterize the reported frequency of hypothyroidism associated with ICI therapy and examine differences by cancer type, treatment regimen, and study completion period. We hypothesized that hypothyroidism would be frequently reported among participants receiving ICIs, particularly in trials evaluating PD-1/PD-L1 inhibitors and combination regimens. The goal of this study was to characterize the reported frequency of hypothyroidism in oncology trials and explore the potential implications for standardized thyroid monitoring, long-term endocrine follow-up, and the incorporation of endocrine irAEs into cancer survivorship planning.

## Review

Materials and methods

Study Design and Reporting Framework

This study was conducted as a registry-based systematic review of clinical trial results and was reported in accordance with the PRISMA 2020 guidelines. All data were obtained from publicly available sources, and no individual-level patient data were accessed; therefore, institutional review board approval was not required. This review was not registered, and a formal protocol was not prepared prior to conducting the study.

Data Source and Search Strategy

Clinical trial data were retrieved from ClinicalTrials.gov, a publicly accessible registry maintained by the U.S. National Library of Medicine. The registry was selected to identify prospectively registered interventional oncology trials with standardized AE reporting. A systematic search strategy was developed using ClinicalTrials.gov. No restrictions were applied regarding trial location, eligibility criteria, sex, age, study phase, study documents, funder type, or date range. No restrictions were applied regarding trial location, eligibility criteria, sex, age, study phase, study documents, funder type, or date range. The condition/disease search term was specified as cancer only. Searches were conducted using intervention/treatment terms related to ICIs. Additional filters included study type (interventional), study results (with results), and status (all statuses). A detailed description of the methods, including search terms, is reported in the appendix. The ClinicalTrials.gov registry was last searched on July 21, 2026.

Eligibility Criteria

Studies were included if they met all of the following criteria: (1) inclusion of all cancer types; (2) enrollment of adult or pediatric populations; (3) randomized or nonrandomized study design; (4) interventional oncology trials of any phase; (5) evaluation of an ICI, either as monotherapy or in combination therapy; and (6) availability of posted study results, including detailed AE data. For trials with multiple intervention arms, study arms were combined when they evaluated the same ICI at different dosing regimens or when the ICI was administered in combination with other therapies, such as targeted therapy, chemotherapy, or radiation therapy. Studies were excluded if they (1) lacked detailed AE reporting, defined as reporting only the overall number of AEs without categorization by AE type, or (2) evaluated non-ICI immunotherapies or non-approved ICIs.

Study Selection Process

Identified records were screened sequentially for relevance based on intervention type and availability of posted results. Trials meeting initial screening criteria underwent a full eligibility assessment using predefined inclusion and exclusion criteria. Reasons for exclusion were documented, and the study selection process was summarized using a PRISMA flow diagram. Screening and eligibility assessment were conducted by a single reviewer (J.W.); no automation tools were used in the selection process.

Data Extraction and Variables

Data were systematically extracted from ClinicalTrials.gov using a standardized data extraction approach. Data were initially downloaded in CSV format and subsequently converted to Microsoft Excel (Microsoft Corp., Redmond, WA) format for data management and further analysis. The complete list of non-duplicate records is presented in the Appendix. For each eligible trial, the following information was collected: ClinicalTrials.gov identifier, study phase, cancer type, treatment regimen, enrollment size, study start and completion dates, and hypothyroidism-related AE data. Hypothyroidism was defined according to the AE terminology reported by each trial, without reclassification or reinterpretation by the investigators. Data extraction was performed by a single reviewer (J.W.) using a standardized data abstraction form. A co-author (X.W.) provided methodological oversight and reviewed the extracted dataset for completeness, consistency, and accuracy, with both processes conducted under the supervision of the senior author (H.R.). Any uncertainties regarding data interpretation or study classification were discussed and resolved by consensus between the reviewer and co-authors.

Outcome Measures

The primary outcome was the trial-level reported frequency of hypothyroidism as an AE among ICI-treated participants. Secondary analyses included the reported frequency of hypothyroidism stratified by cancer type, treatment regimen (monotherapy versus combination therapy), and temporal trends based on study completion dates. Serious AEs were identified as reported by the original trial investigators in accordance with regulatory definitions. Per ICH (International Council for Harmonisation) E2A/E6 guidelines, a serious AE is defined as any AE that results in death, is life-threatening, requires or prolongs hospitalization, results in persistent or significant disability or incapacity, causes a congenital anomaly or birth defect, or is otherwise considered medically significant such that intervention is required to prevent one of these outcomes.

Risk of Bias, Reporting Bias, and Certainty Assessment

A formal risk-of-bias assessment using a standardized tool, such as Cochrane RoB 2, was not performed, since this study reports registry-listed AE frequencies rather than comparing treatment efficacy or safety, the purpose such tools are built for. Reporting bias from trials that ran but never posted results could not be assessed either; this is noted instead as a limitation. Certainty of evidence was not graded using a system like GRADE (Grading of Recommendations, Assessment, Development, and Evaluations), since the pooled trial-reported proportions here are meant as descriptive summaries of what trials reported rather than as causal effect estimates.

Data Synthesis and Analysis

Data were summarized descriptively. Reported proportions were calculated using reported numerators and denominators from individual trials. Due to heterogeneity in trial design, populations, and reporting practices, no quantitative meta-analysis was performed. Temporal comparisons were conducted by stratifying studies based on the median completion date in chronological order. For analysis restricted to the same treatment and cancer type, including subgroup and temporal trend analyses, data were combined, and reported AE frequency was estimated by pooling event counts and total numbers of treated patients across eligible study groups. For each outcome, the number of patients experiencing the event and the total number of patients at risk were summed across studies to derive a pooled proportion. Ninety-five percent confidence intervals (CIs) for pooled trial-reported proportions were calculated using the exact binomial (Clopper-Pearson) method to account for sparse events and small sample sizes in individual groups. Pooled estimates were interpreted as descriptive, trial-level summaries and were not intended to represent patient-level causal effects. No sensitivity analyses were conducted, given the primarily descriptive aims of this review. These CIs describe uncertainty around the aggregated event proportions but do not account for between-trial heterogeneity and should not be interpreted as meta-analytic CIs.

Software and Data Handling

All data organization and statistical calculations were performed using Microsoft Excel. No custom analytic software or external code repositories were utilized. The extracted dataset used for this analysis is available from the corresponding author upon reasonable request.

Results

Search and Study Selection

The search retrieved 164 records. After removal of 67 duplicate records, 38 non-immunotherapy records, and three records without AE reports, 56 unique records underwent screening. Two studies (NCT03257722 and NCT03197389) were excluded because detailed AE data were unavailable, leaving 54 reports for eligibility assessment. Of these, 12 were excluded: three evaluated other immunotherapies and nine evaluated unapproved ICIs. Examples of excluded investigational agents included bifunctional proteins targeting TGF-β and PD-L1 (NCT04501094, NCT04327986, NCT04574583, and NCT03315871), an anti-NKG2A antibody (NCT04590963), and an investigational oral small-molecule PD-L1 inhibitor (NCT04629339). After applying all eligibility criteria, 42 studies were included in the final analysis, as summarized in the PRISMA flow diagram (Figure 1). The study start dates ranged from August 2006 to June 28, 2023, with completion dates spanning from October 2013 to September 2030 (estimated), reflecting both mature clinical trial datasets and ongoing investigations.

**Figure 1:**
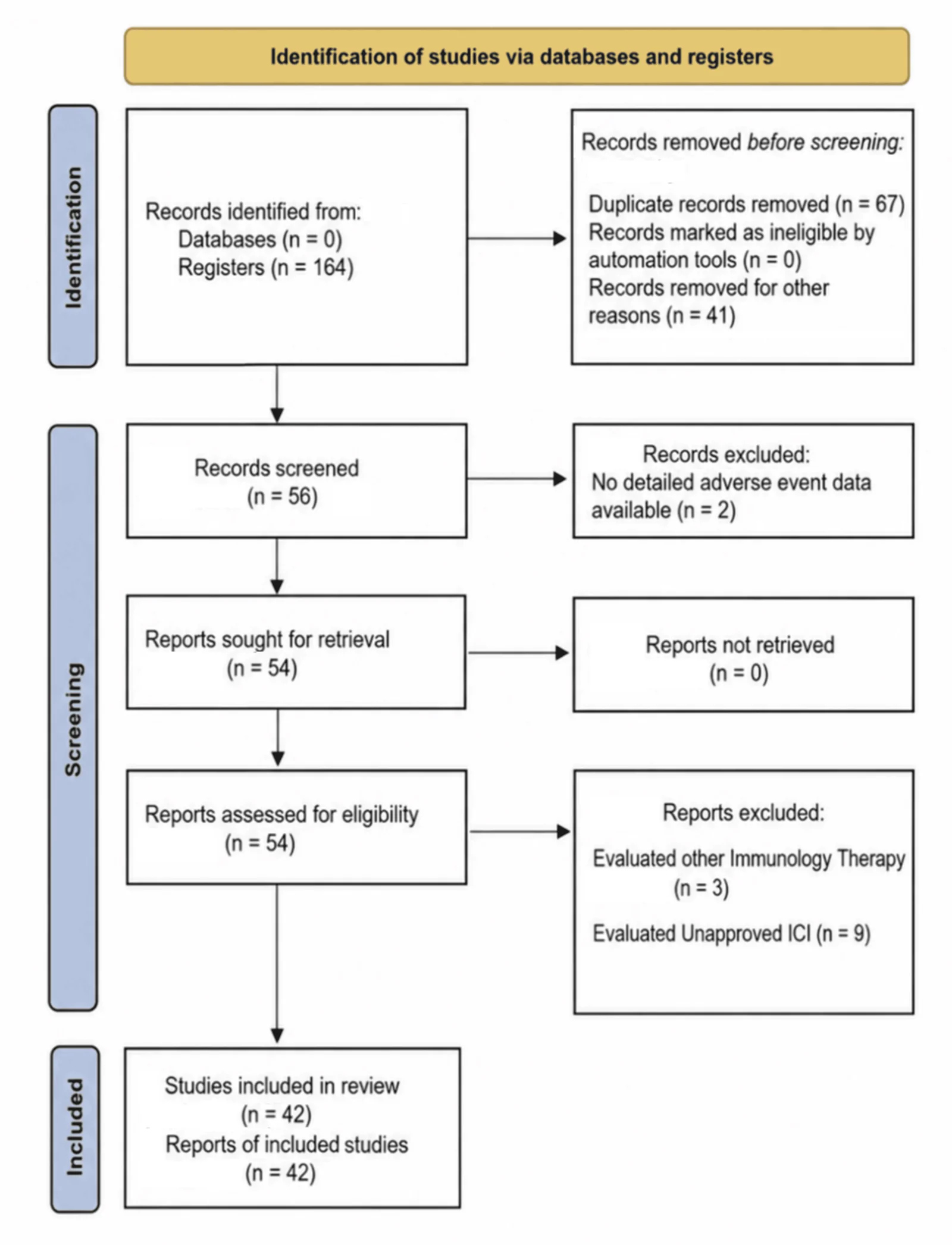
PRISMA 2020 flow diagram of study selection ClinicalTrials.gov was systematically searched to identify interventional oncology trials evaluating ICIs with posted results. Trials were screened, assessed for eligibility, and included according to predefined criteria aligned with the PRISMA 2020 guidelines. ICI: Immune checkpoint inhibitors; PRISMA: Preferred Reporting Items for Systematic Reviews and Meta-Analyses.

Characteristics of Included Trials

The final dataset included studies evaluating a range of approved ICIs targeting the PD-1, PD-L1, and CTLA-4 pathways, administered either as monotherapy or in combination with other anticancer treatments. The included trials represented diverse patient populations, tumor types, and clinical settings, thereby providing a descriptive assessment of treatment-related AEs across the contemporary ICI landscape. Study characteristics, including trial phase, treatment regimen, cancer type, sample size, and follow-up duration, are summarized in Table 1. Across the included studies, substantial variability was observed in study design and enrollment size, reflecting differences in therapeutic indications, development stages, and treatment strategies. This heterogeneity allowed inclusion of multiple clinical contexts but limited the comparability of pooled estimates.

**Table 1:** Baseline characteristics of included clinical trials Data were extracted from ClinicalTrials.gov using the corresponding National Clinical Trial (NCT) identifiers listed in the table [12]. GBM: Glioblastoma; HNSCC: Head and neck squamous cell carcinoma; MEL: Melanoma; NA: Not available; NSCLC: Non-small cell lung cancer; PD-1: Programmed cell death protein 1; PD-L1: Programmed cell death ligand 1; PD-(L)1: PD-1/PD-L1; RCC: Renal cell carcinoma; SCLC: Small cell lung cancer; UC: Urothelial carcinoma.

Clinical Trial ID	Indication	Intervention of ICI	Study Start (Actual)	Study Completion (Actual/Estimated)	Enrollment (Actual)	Phase
NCT03313804	NSCLC HNSCC	Nivolumab ,Pembrolizumab, Atezolizumab	10/26/2017	2/16/2026	76	2
NCT05846646	NSCLC RCC	Pembrolizumab or Nivolumab	6/28/2023	9/16/2024	6	2
NCT02973789	NSCLC	Nivolumab, Pembrolizumab, or Atezolizumab	12/2016	10/9/2024	291	3
NCT04020185	Solid tumor	Anti-PD-(L)1-ICI	9/23/2019	9/15/2023	40	1/2
NCT04577807	MEL	Anti-PD-1-ICI	11/17/2020	11/15/2024	27	2
NCT04538950	Cancer type not defined	ICI	8/14/2020	5/19/2022	6	1/2
NCT04637594	UC	Anti-PD-(L)1-ICI	5/27/2022	09/2030	3	3
NCT03388632	Solid tumor	Nivolumab + Ipilimumab	2/5/2018	5/8/2025	31	1
NCT04508725	RCC	ICI	12/5/2020	11/30/2023	22	NA
NCT04376502	Solid tumor	ICI	4/8/2020	10/4/2023	12	NA
NCT02971956	Esophageal cancer	Pembrolizumab	1/18/2017	5/13/2020	49	2
NCT02903914	Solid tumors	Pembrolizumab	9/14/2016	8/15/2022	260	1
NCT03878979	HNSCC	Nivolumab	7/8/2019	10/17/2023	26	2
NCT05308901	Uveal MEL	Pembrolizumab + Lenvatinib	8/2/2022	06/2027	6	2
NCT03493932	GBM	Nivolumab + BMS-986017	9/24/2018	6/27/2023	21	1
NCT03041311	SCLC	Atezolizumab	6/29/2017	10/29/2020	107	2
NCT04479241	GBM	Pembrolizumab + Lerapolturev	10/21/2020	6/21/2024	25	2
NCT02664181	NSCLC	Nivolumab + THU + Decitabine	6/6/2017	5/19/2024	13	2
NCT03025035	Breast cancer	Pembrolizumab + Olaparib	9/10/2017	11/21/2025	14	2
NCT03789110	Breast cancer	Nivolumab + Ipilimumab	3/8/2019	2/28/2027	30	2
NCT04225039	GBM	INCMGA00012(anti-PD-1) + INCAGN01876 +RT	6/23/2020	4/30/2029	39	2
NCT03406858	Prostate cancer	Pembrolizumab ± T cells	6/7/2018	11/7/2022	15	2
NCT03331562	Pancreatic cancer	Pembrolizumab + Paricalcitol	12/27/2017	7/10/2020	24	2
NCT02608684	Ovarian cancer	Pembrolizumab + Cisplatin + Gemcitabine	2/8/2016	3/7/2022	21	2
NCT05041062	Peritoneal mesothelioma	Nivolumab + Ipilimumab	12/1/2021	4/13/2023	2	2
NCT03761914	Solid tumor	Galinpepimut-S + Pembrolizumab	9/30/2019	10/27/2022	26	1/2
NCT03792347	Squamous cell carcinoma of esophagus	Pembrolizumab	1/21/2019	6/17/2020	20	1
NCT04045613	UC	Atezolizumab + Derazantinib	8/2/2019	10/4/2022	95	1/2
NCT02224781	MEL	Nivolumab + Ipilimumab	9/8/2015	12/19/2026	267	3
NCT04696055	Liver cancer	Pembrolizumab + Regorafenib	2/3/2021	4/23/2024	95	2
NCT02879695	Leukemia	Nivolumab + Ipilimumab + Blinatumomab	10/25/2017	7/18/2026	28	1
NCT03307785	Solid tumor	TSR-042 combination (anti-PD-1)	10/12/2017	12/11/2024	60	1
NCT03035890	NSCLC	Nivolumab, Pembrolizumab, or Atezolizumab	1/23/2017	6/30/2022	35	NA
NCT01642004	NSCLC	Nivolumab	10/16/2012	8/16/2021	272	3
NCT01866319	MEL	Pembrolizumab	8/28/2013	6/3/2019	834	3
NCT02008227	NSCLC	Atezolizumab	3/11/2014	1/9/2019	1225	3
NCT01905657	NSCLC	Pembrolizumab	8/9/2013	9/30/2020	1034	2/3
NCT02366143	NSCLC	Atezolizumab + Paclitaxel + Carboplatin	3/31/2015	12/7/2020	1202	3
NCT02178722	Solid tumor	Pembrolizumab + Epacadostat	7/17/2014	11/6/2020	444	1/2
NCT00324155	MEL	Ipilimumab + Dacarbazine	08/2006	10/2013	681	3
NCT02477826	NSCLC	Nivolumab, or Nivolumab + Ipilimumab, or Nivolumab + Platinum Doublet Chemotherapy	8/5/2015	10/25/2024	2747	3
NCT02684006	RCC	Avelumab + Axitinib	3/23/2016	6/26/2024	886	3

Reporting and Definition of Hypothyroidism

Hypothyroidism events of any reported grade were extracted using the sponsor-reported MedDRA preferred term “hypothyroidism” in ClinicalTrials.gov safety results. The registry records generally did not provide diagnostic criteria or sufficient clinical detail to determine whether an event represented primary hypothyroidism, the hypothyroid phase of destructive thyroiditis, or permanent thyroid dysfunction. We therefore analyzed only events explicitly reported as hypothyroidism and did not reclassify related thyroid events. Differences in diagnostic, grading, and attribution practices across trials were considered when interpreting the pooled trial-reported proportions.

Rate of ICI-Associated Hypothyroidism Across Trials

Among the 42 eligible trials spanning phases 1 through 3, 27 studies (64.29%) reported hypothyroidism as an AE. Across cancer types, renal cell carcinoma had the highest trial-reported frequency of hypothyroidism at 31.6% (95% CI, 27.4%-36.1%), followed by ovarian cancer at 27.8% (95% CI, 12.5%-50.9%) and melanoma at 15.6% (95% CI, 13.2%-18.4%). In contrast, esophageal cancer demonstrated the lowest reported frequency of hypothyroidism at 6.1% (95% CI, 2.1%-16.5%) (Figure 2). These pooled estimates were calculated from reported hypothyroidism events and treated-patient denominators across studies with varying designs, populations, and treatment regimens. Therefore, the findings should be interpreted as trial-reported frequencies rather than direct measures of disease-specific risk.

**Figure 2:**
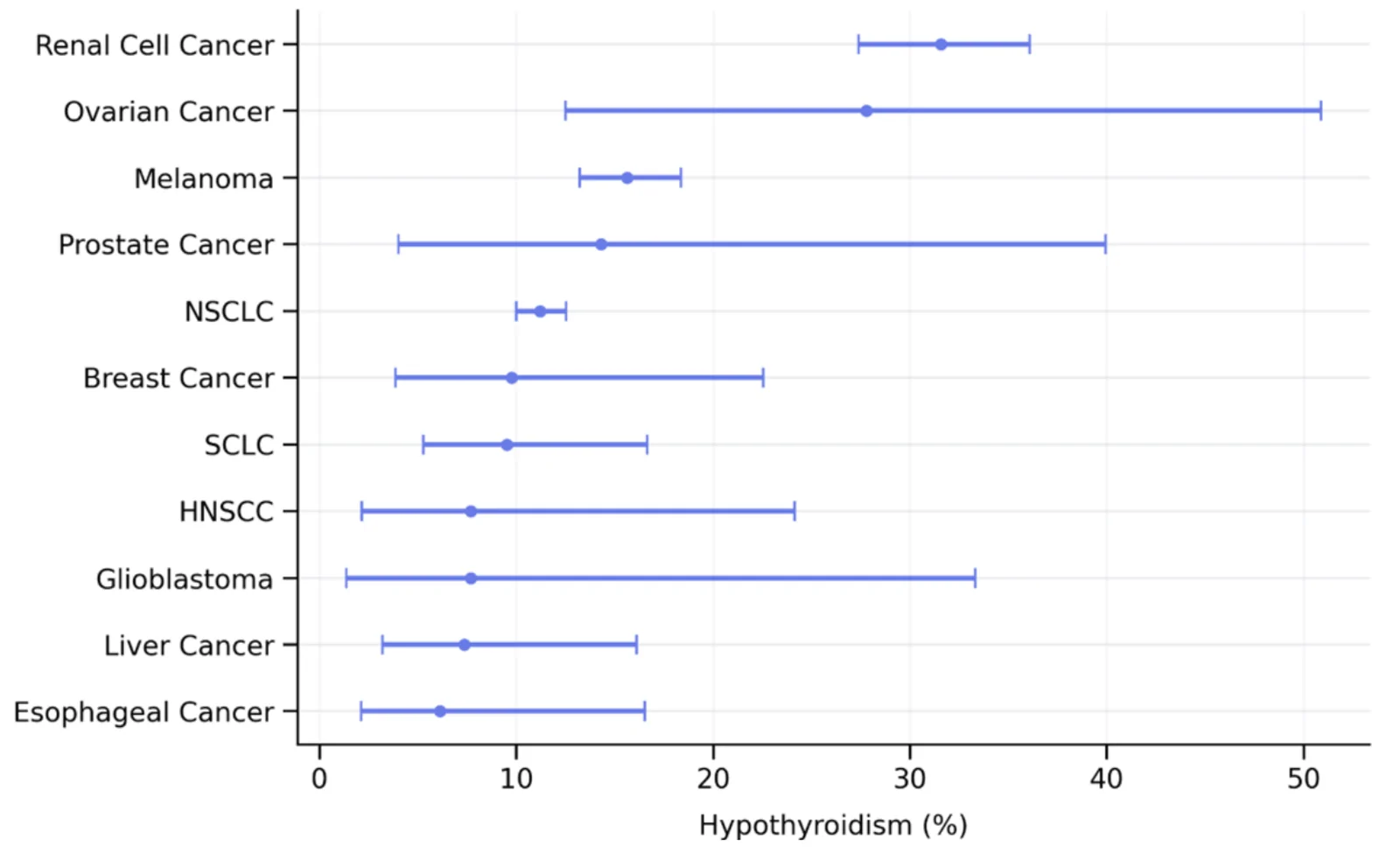
Frequency of hypothyroidism across cancer types in patients treated with immune checkpoint inhibitors (ICIs) Point estimates represent the trial-reported proportion of patients with hypothyroidism for each cancer type, and horizontal error bars indicate 95% confidence intervals (CI). The highest reported proportions were observed in renal cell cancer (31.6%; 95% CI, 27.4–36.1; N = 434) and ovarian cancer (27.8%; 95% CI, 12.5–50.9; N = 18), whereas lower reported proportions were observed in glioblastoma (7.7%; 95% CI, 1.4–33.3; N = 13), head and neck squamous cell carcinoma (HNSCC) (7.7%; 95% CI, 2.1–24.1; N = 26), liver cancer (7.4%; 95% CI, 3.2–16.1; N = 68), and esophageal cancer (6.1%; 95% CI, 2.1–16.5; N = 49). Intermediate reported proportions were observed in melanoma (15.6%; 95% CI, 13.2–18.4; N = 756), prostate cancer (14.3%; 95% CI, 4.0–39.9; N = 14), non-small cell lung cancer (NSCLC) (11.2%; 95% CI, 10.0–12.5; N = 2385), breast cancer (9.8%; 95% CI, 3.9–22.6; N = 41), and small cell lung cancer (9.5%; 95% CI, 5.3–16.7; N = 105). Image credit: Created by the authors using Microsoft Excel (Microsoft Corp., Redmond, WA).

By treatment regimen, anti-PD-1 combination therapies showed the highest reported proportion (Figure 3). The highest combined frequency was observed with nivolumab plus ipilimumab, at 18.8% (95% CI, 16.4%-21.4%); pembrolizumab plus lenvatinib also registered a high value. However, as it reflects only one of six patients, it is reported here as a raw count rather than as a stable rate. Among monotherapies, pembrolizumab was associated with a higher trial-reported rate of hypothyroidism at 9.1% (95% CI, 7.7%-10.8%) than nivolumab (Figure 3).

**Figure 3:**
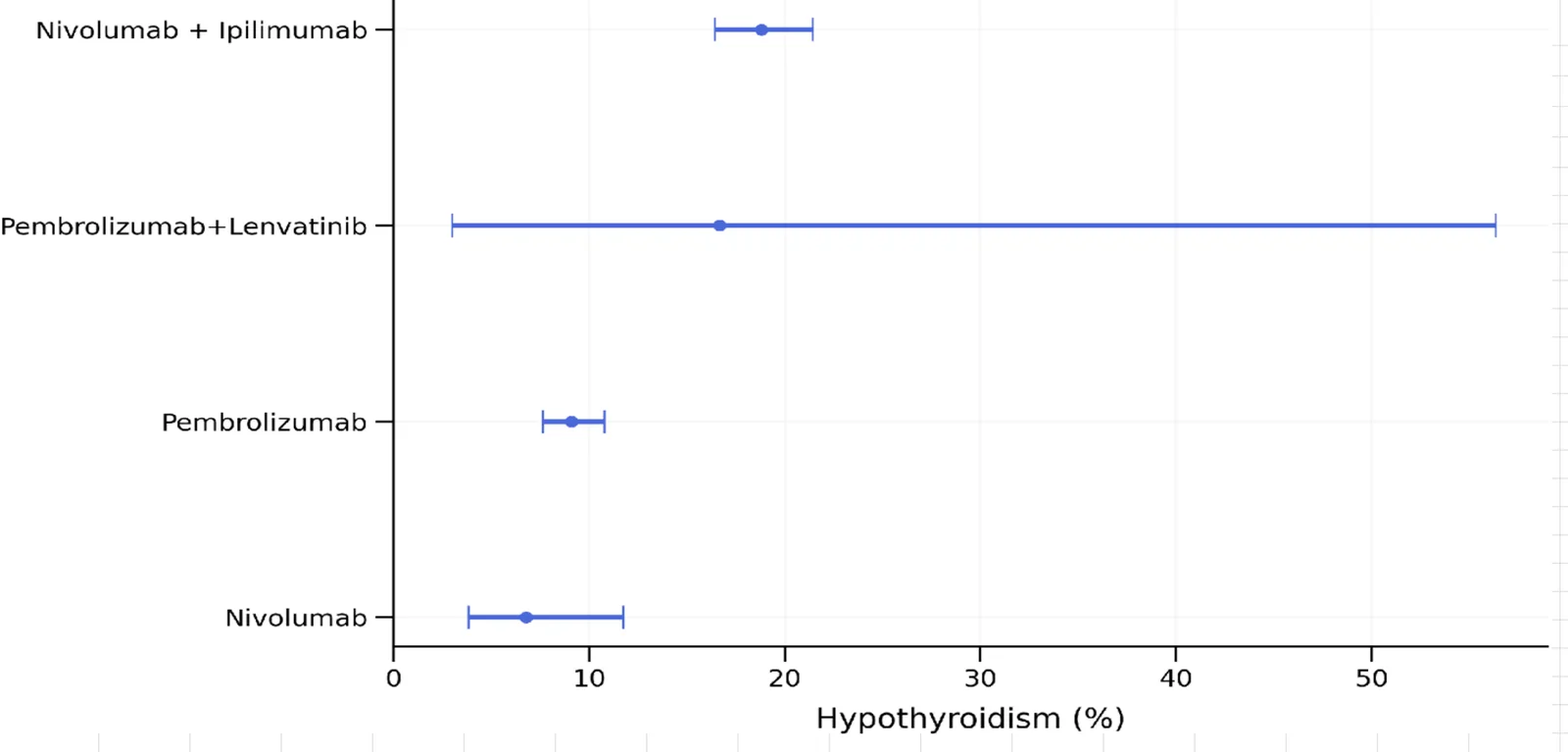
Frequency of hypothyroidism associated with immune checkpoint inhibitor (ICI) therapies Point estimates represent the trial-reported proportion of patients with hypothyroidism for each treatment regimen across the 27 studies that reported hypothyroidism as an adverse event, and horizontal error bars indicate 95% confidence intervals (CI). The regimens included nivolumab + ipilimumab (18.8%; 95% CI, 16.4–21.4; N = 941), pembrolizumab + lenvatinib (16.7%; 95% CI, 3.0–56.4; N = 6), pembrolizumab (9.1%; 95% CI, 7.7–10.8; N = 1307), and nivolumab (6.8%; 95% CI, 3.8–11.8; N = 162). Confidence intervals reflect variability in estimates, with wider intervals observed in studies with smaller sample sizes. Image credit: Created by the authors using Microsoft Excel (Microsoft Corp., Redmond, WA).

NSCLC Subgroup Analyses

An exploratory subgroup analysis was conducted for NSCLC. Six studies enrolled patients with non-small cell lung cancer (NSCLC); one multi-ICI study was excluded from subgroup analysis. NSCLC was selected because it represented the largest disease category in the dataset and a major area of ICI utilization. In addition, lung cancer remains the leading cause of cancer-related mortality worldwide, contributing around 1.8 million deaths annually [13]. As NSCLC comprises around 85% of all lung cancers, focusing specifically on this subtype allows our findings to evaluate ICI-associated hypothyroidism within the predominant lung cancer population treated with immunotherapy [14]. Among the remaining five NSCLC studies, the highest observed frequency was seen with nivolumab plus ipilimumab, followed by atezolizumab and pembrolizumab. The remaining two nivolumab monotherapy studies reported a proportion of 7.69% and 6.10%, respectively (Figure 4). This analysis was undertaken to provide additional context regarding hypothyroidism frequency within the most extensively represented cancer type; however, it was not a prespecified primary outcome of the study.

Temporal Trends

To explore potential changes in the reporting of hypothyroidism over time, studies were grouped chronologically according to the median study completion date (October 17, 2023). Among the 27 studies reporting hypothyroidism outcomes, the earliest 13 studies demonstrated a trial-reported hypothyroidism frequency of 9.11%, whereas the most recent 14 studies reported a higher frequency of 19.83%. This finding suggests a possible upward temporal trend in the reporting of ICI-associated hypothyroidism. However, the temporal analyses were exploratory and descriptive in nature and should be interpreted with caution. Nevertheless, changes over time in trial design, patient populations, treatment practices, follow-up duration, and the recognition and reporting of irAEs may have contributed to the observed differences. As such, these results should not be interpreted as definitive evidence of a true increase in risk but rather as reflecting evolving clinical and research practices that may influence estimates of ICI-associated hypothyroidism.

**Figure 4:**
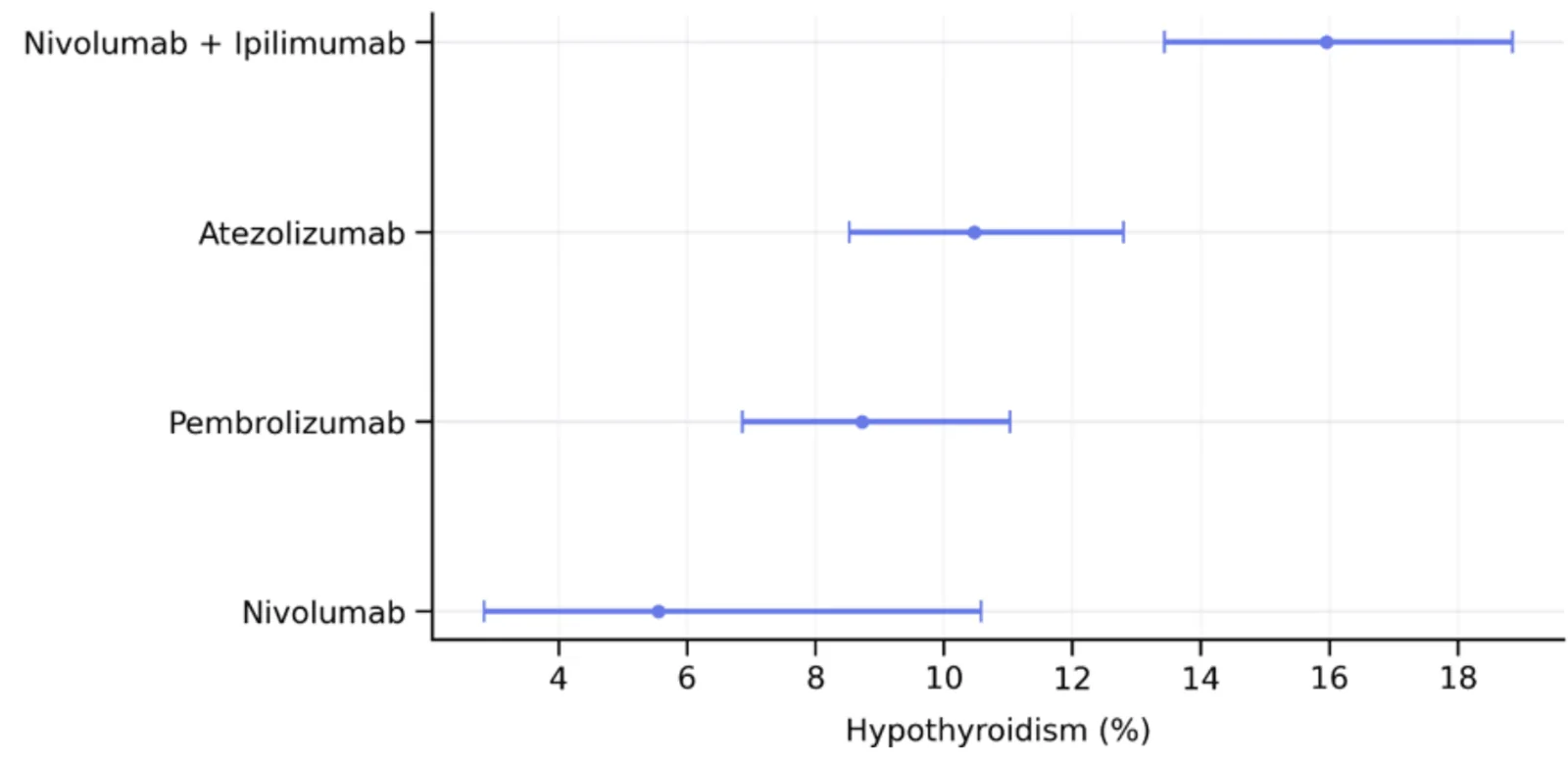
Frequency of hypothyroidism in NSCLC patients receiving ICI monotherapy or combination therapy Point estimates represent the trial-reported proportion of patients with hypothyroidism for each treatment regimen, and horizontal error bars indicate 95% confidence intervals (CI). The reported proportion of hypothyroidism was 16.0% (95% CI, 13.4–18.8; N = 702) for nivolumab plus ipilimumab, 10.5% (95% CI, 8.5–12.8; N = 793) for atezolizumab, 8.7% (95% CI, 6.9–11.0; N = 711) for pembrolizumab, and 5.6% (95% CI, 2.8–10.6; N = 144) for nivolumab. Image credit: Created by the authors using Microsoft Excel (Microsoft Corp., Redmond, WA).

Serious AEs

Among the 27 studies reporting hypothyroidism, six studies (22.22%) classified hypothyroidism as a serious adverse event (SAE). The occurrence of serious hypothyroidism illustrates an increased challenge for patients, caregivers, and the health care system. Severe hypothyroidism can cause myxedema coma and impaired cognition, outcomes that may necessitate emergent hospitalization and intervention. Even cases that do not meet the formal threshold for an SAE carry meaningful consequences like lifelong hormone replacement therapy [15].

Discussion

In this PRISMA-aligned systematic review of ClinicalTrials.gov-posted results, hypothyroidism emerged as a common irAE across phase 1-3 ICI trials, reported in nearly two-thirds of included studies. These findings reinforce prior evidence that thyroid dysfunction is the most frequent endocrine toxicity of ICIs and extend that picture by demonstrating its growing relevance across tumor types, treatment combinations, and time [1-3,5].

Our analysis demonstrates substantial heterogeneity in hypothyroidism-reported frequency by cancer type and treatment regimen. Renal cell carcinoma trials showed the highest observed frequency of hypothyroidism, a finding consistent with previous reports suggesting that tumor type and immune microenvironment may influence susceptibility to endocrine irAEs [6,11]. Host autoimmune predisposition may also contribute to differences in thyroid toxicity across trial populations. A 2025 systematic review and meta-analysis reported a pooled thyroid irAE incidence of 66.7% among patients with baseline-positive thyroid antibodies, suggesting that antibody status warrants further investigation as a potential risk marker [16]. This study included broad thyroid irAE outcomes, so its findings do not establish thyroid antibodies as a definitive predictor for hypothyroidism. Even so, it remains relevant because it identifies a patient-level factor unavailable in ClinicalTrials.gov that may partly explain between-trial variation in the reported hypothyroidism frequencies.

Combination ICI regimens, particularly nivolumab plus ipilimumab, had higher descriptive pooled proportions of hypothyroidism events than monotherapy, which aligns with mechanistic and clinical evidence indicating that dual immune checkpoint blockade leads to broader immune activation and a higher cumulative burden of immune-related toxicity [17,18]. More recently, a 2025 systematic review and meta-analysis of 40 randomized controlled trials involving 12,376 patients found an increased risk of hypothyroidism following ICI treatment and greater thyroid dysfunction with combination ICI therapy than with monotherapy [19]. That study calculated comparative risk estimates, whereas our registry analysis calculated descriptive pooled proportions. The numerical estimates are therefore not directly comparable, but both studies showed the same general pattern of greater thyroid toxicity with combination ICI regimens. This idea is further supported by a same-trial comparison: in a phase 3 study (NCT02477826), patients randomized to nivolumab and ipilimumab experienced hypothyroidism (16.0%) around twice the proportion observed in patients randomized to nivolumab monotherapy (7.69%). Since both treatment groups came from the same trial population, this comparison avoids the cross-trial bias. This provides a cleaner signal that treatment allocation to the combination arm was associated with an increased observation of hypothyroidism, rather than reflecting differences between study populations.

Prior literature suggests that combination ICI therapy blocks multiple immune regulatory pathways simultaneously, producing greater T-cell activation and a broader loss of immune tolerance [20]. This may increase the likelihood that hormone-producing capacity is lost and, consequently, be associated with the development of hypothyroidism. While this study did not directly assess immune mechanisms, these published findings offer a plausible biological explanation for the greater occurrence of hypothyroidism observed with combination regimens. Notably, pembrolizumab monotherapy demonstrated a higher reported proportion of hypothyroidism compared with nivolumab in this registry-based analysis. Cross-trial comparisons must be interpreted with caution, given differences in study design and patient populations. Still, this pattern is consistent with meta-analyses identifying differential endocrine toxicity profiles among individual ICIs [8]. The pooled proportions observed in this analysis were higher than the regimen-specific ranges reported in the broader literature, where hypothyroidism occurs in roughly 3.9%-8.5% of patients receiving PD-1/PD-L1 monotherapy and 10.2%-16.4% with combination therapy. Our combination estimate, in particular, sits above that published ceiling [11]. Possible explanations include differences in case definitions, follow-up, regimen composition, cancer type, and reporting thresholds. These findings underscore the importance of agent-specific risk assessment and individualized monitoring strategies in clinical practice.

One of the most striking observations in this analysis is the apparent increase in reported hypothyroidism occurrence in more recent trials compared to earlier ones. This trend may reflect multiple converging factors: more frequent use of combination regimens, longer treatment durations, improved recognition and reporting of endocrine AEs, and the expansion of ICI use into patient populations with greater survival potential [9,10]. Regardless of whether this reflects rising risk per patient or simply broader use of overlapping contributors, the growing scale of ICI exposure means that long-term endocrine burden associated with these therapies is likely to intensify.More than one-fifth of studies reporting hypothyroidism classified it as an SAE. Although hypothyroidism is often perceived as a manageable toxicity, severe cases can necessitate hospitalization and urgent intervention, and even milder forms carry lasting consequences: permanent hormone replacement, chronic healthcare utilization, and quality-of-life impacts that extend well into survivorship [21,22]. These findings challenge the notion that endocrine irAEs are uniformly mild.

Limitations and future directions

This study has several important limitations. Registry-reported AE data are subject to underreporting, variability in severity grading, and inconsistent attribution to the study drug across trials. In addition, hypothyroidism is itself a heterogeneous endpoint across trials, where many recorded cases represent the terminal phase of a thyroiditis or thyrotoxicosis-to-hypothyroidism trajectory. Trials also differed in whether they captured the transient thyrotoxic phase; therefore, we extracted each trial’s coded term without harmonizing it across the biphasic course. Further, differences in follow-up duration and the potential for residual overlap among registry records may have affected the comparability and accuracy of reported frequencies across studies. Moreover, individual patient-level data, including baseline thyroid function, thyroid autoantibody status, and the duration of thyroid dysfunction, were not available, preventing assessment of host predisposition as a potential source of trial variation. Heterogeneity in trial design and reporting practices also precluded formal meta-analysis. Adverse-event grading mostly relied on Common Terminology Criteria for Adverse Events (CTCAE) criteria, yet inter-investigator variability, differences in the interpretation of endocrine toxicity definitions, and variation in monitoring frequency may have contributed to inconsistencies across studies.Delays between symptom onset, the detection of laboratory abnormalities, clinical diagnosis, and documentation may also have affected the accuracy of reported frequency estimates and temporal analyses. Other biomarker data, including longitudinal hormone measurements, inflammatory markers, and genomic or immunologic correlates, were frequently incomplete or inconsistently reported.This limited mechanistic understanding and the identification of susceptible patient populations.

Long-term surveillance was also often insufficient, potentially leading to underrecognition of delayed endocrine dysfunction, persistent hormone deficiencies, or late recovery patterns. Diagnostic masking presents another clinical challenge because endocrine AEs frequently cause nonspecific symptoms, such as fatigue, weight changes, or weakness, that overlap with manifestations of advanced malignancy, concurrent therapies, or preexisting comorbidities.Beyond the biphasic course described above, variability in the latency, progression, and recovery of immune-mediated tissue destruction was not adequately captured by registry-reported adverse-event terms.Interpretation may also have been complicated by central-axis confounding, particularly when abnormalities involving the hypothalamic-pituitary axis mimicked or influenced peripheral thyroid dysfunction. More broadly, the development of endocrine toxicities is likely shaped by multiple interacting risk factors, including patient characteristics, preexisting autoimmune susceptibility, tumor biology, treatment exposure, concomitant medications, and temporal changes in immune activation. Despite these limitations, the registry-based design allowed descriptive comparisons across multiple cancer types, treatment regimens, and study periods. Finally, screening, eligibility assessment, and data extraction were conducted by one primary reviewer. A coauthor reviewed the extracted dataset for completeness, consistency, and accuracy under the supervision of the senior author. Independent duplicate screening and extraction were not performed, which may have increased the risk of selection and extraction errors.

Due to the limitations of this registry-based analysis, several questions remain unresolved and should motivate further investigation. One such question is why certain cancer types treated with ICIs show higher reported proportions of hypothyroidism than others. One possible contributor is host autoimmune predisposition. Baseline thyroid autoantibody status has been investigated as a potential risk marker for thyroid irAEs, although its relationship with hypothyroidism specifically remains uncertain. We could not assess this association without patient-level serology, highlighting the need for studies that examine whether preexisting thyroid autoimmunity contributes to differences in hypothyroidism risk across patients. Further clinical trials comparing thyroid surveillance schedules are also needed to inform standardized monitoring guidelines. Finally, health economics research quantifying the population-level costs of ICI-associated hypothyroidism, including long-term hormone replacement, laboratory monitoring, and specialist referrals, could help determine the potential value of proactive surveillance strategies.

## Conclusions

This registry-based systematic review demonstrates that ICI-associated hypothyroidism is commonly reported across clinical trials, with substantial variation across cancer types, treatment regimens, and study periods. The highest pooled reported proportion by cancer type was observed in renal cell carcinoma, while nivolumab plus ipilimumab had the highest pooled proportion among the evaluated treatment regimens. More recently completed trials had a higher descriptive pooled proportion of reported hypothyroidism, although differences in trial composition and reporting practices prevent this finding from establishing a temporal increase in patient-level risk. As immunotherapy reaches more patients at earlier disease stages with longer expected survival, the endocrine consequences of these treatments may represent an increasing component of cancer survivorship care. Standardized thyroid monitoring, long-term endocrine follow-up, and integration of endocrine irAE tracking into survivorship frameworks may be warranted and should be addressed in future guidelines. Future studies should investigate patient-level predictors of ICI-associated hypothyroidism, including baseline thyroid autoimmunity, while also evaluating optimal surveillance strategies and the long-term health system burden of this toxicity. These efforts would help clarify which patients are at greatest risk and how thyroid monitoring can be incorporated more effectively into survivorship care.

## Appendices

Search strategy and consideration of inclusion and exclusion criteria

We followed the Preferred Reporting Items for PRISMA guidelines to develop the search strategy using the ClinicalTrials.gov registry. Specifically, the search terms included "immune checkpoint inhibitor," "immune checkpoint inhibitors," "ICI," and "ICIs," encompassing agents targeting PD-1, PD-L1, CTLA-4, and other immune checkpoint pathways. Four separate searches were conducted using these terms, and the resulting records were combined into a single dataset.

Additional filters were applied to identify relevant studies, including study type (interventional), availability of study results (with results), and recruitment status (all statuses). To enable evaluation of adverse event outcomes, only trials with posted safety results were considered eligible for screening.

Duplicate records were identified using the unique National Clinical Trial (NCT) number assigned to each study, and duplicate entries were removed before screening. Records were also removed if they evaluated non-immunotherapy alone, including targeted therapies, chemotherapy, antibody-drug conjugates (ADCs), hormonal therapies, or radiation therapy. However, studies in which an ICI was administered in combination with these treatment modalities were retained and further evaluated during the study selection process. Reports evaluating non-ICI immunotherapies or unapproved immune checkpoint inhibitors were excluded. The unapproved ICIs included two categories: (1) investigational ICIs targeting non-approved checkpoint pathways, such as NKG2A, TIGIT, TIM-3, BTLA, or VISTA; and (2) investigational agents targeting approved checkpoint pathways (PD-1, PD-L1, CTLA-4, or LAG-3) engineered to incorporate additional immunomodulatory functions or novel technical strategies. For example, bintrafusp alfa was excluded because it is an investigational PD-L1/TGF-β bifunctional fusion protein rather than an approved ICI.

Supplementary results

The complete list of non-duplicate records, along with the corresponding removal, inclusion, or exclusion reasons provided in Column B, is presented in the following table.

**Table 2:** Characteristics and hypothyroidism adverse events reported across included immune checkpoint inhibitor clinical trials Hypothyroidism events are reported according to the terminology provided in the corresponding ClinicalTrials.gov record. Trial information and adverse-event data were obtained from ClinicalTrials.gov. ICI: Immune checkpoint inhibitor; CTLA-4: Cytotoxic T-lymphocyte–associated protein 4; PD-1: Programmed cell death protein 1; PD-L1: Programmed death-ligand 1.

Reason for Removal/Exclusion/Inclusion	Study Title	Study Results	Conditions	Interventions	Sex	Age	Phases	Enrollment	Study Type	Start Date	Primary Completion Date	Completion Date
ICI + RT	Priming Immunotherapy in Advanced Disease With Radiation	YES	Non-Small Cell Lung Cancer | Squamous Cell Carcinoma of the Head and Neck	DRUG: Immune checkpoint inhibitor | RADIATION: Radiation Therapy	ALL	ADULT, OLDER_ADULT	PHASE 2	76	INTERVENTIONAL	26/10/2017	16/02/2024	16/02/2026
ICI + TTF	Effect of Tumor Treating Fields (TTFields) (150 kHz) Concurrent With Standard of Care Therapies for Treatment of Stage 4 Non-Small Cell Lung Cancer (NSCLC) Following Platinum Failure (LUNAR)	YES	Non-Small Cell Lung Cancer | NSCLC	DEVICE: NovoTTF-200T | DRUG: Immune checkpoint inhibitors or docetaxel	ALL	ADULT, OLDER_ADULT	PHASE 3	291	INTERVENTIONAL	2016/12	24/09/2024	09/10/2024
ICI	LUMINOS-102: Lerapolturev With or Without Immune Checkpoint Blockade in Advanced PD-1 Refractory Melanoma	YES	Melanoma	BIOLOGICAL: Lerapolturev | BIOLOGICAL: Anti-PD-1 Checkpoint Inhibitor	ALL	ADULT, OLDER_ADULT	PHASE 2	27	INTERVENTIONAL	17/11/2020	15/11/2024	15/11/2024
ICI + IL-15	Recombinant Interleukin-15 in Combination With Checkpoint Inhibitors Nivolumab and Ipilimumab in People With Refractory Cancers	YES	Metastatic Solid Tumors | Treatment-Refractory Cancers	DRUG: rhIL-15 | DRUG: Ipilimumab | DRUG: Nivolumab | PROCEDURE: Tumor biopsies | DIAGNOSTIC_TEST: EKG | DIAGNOSTIC_TEST: ECHO | DIAGNOSTIC_TEST: CT Scan	ALL	ADULT, OLDER_ADULT	PHASE 1	31	INTERVENTIONAL	05/02/2018	27/11/2024	08/05/2025
Pembrolizumab	Pembrolizumab in Refractory Advanced Esophageal Cancer	YES	Esophageal Cancer | Squamous Cell Esophagus Cancer | Adenocarcinoma Esophagus	DRUG: Pembrolizumab	ALL	ADULT, OLDER_ADULT	PHASE 2	49	INTERVENTIONAL	18/01/2017	23/07/2019	13/05/2020
Anti-EGFR ab + TKI	Dual Inhibition of EGFR With Afatinib and Cetuximab in the Treatment of Advanced Squamous Cell Cancers of the Head and Neck	YES	Squamous Cell Cancers of the Head and Neck	DRUG: cetuximab | DRUG: afatinib	ALL	ADULT, OLDER_ADULT	PHASE 2	50	INTERVENTIONAL	24/03/2017	18/01/2025	18/01/2026
Pembrolizumab	Arginase Inhibitor INCB001158 as a Single Agent and in Combination With Immune Checkpoint Therapy in Patients With Advanced/Metastatic Solid Tumors	YES	Metastatic Cancer | Solid Tumors | Colorectal Cancer (CRC) | Gastric Cancer | Head and Neck Cancer | Lung Cancer | Renal Cell Carcinoma (RCC) | Bladder Cancer | UC (Urothelial Cancer) | Mesothelioma	DRUG: INCB001158 | DRUG: Pembrolizumab	ALL	ADULT, OLDER_ADULT	PHASE 1	260	INTERVENTIONAL	14/09/2016	30/04/2020	15/08/2022
A potent small-molecule inhibitor of PD-L1	Study of INCB086550 in Select Solid Tumors	YES	Non-Small Cell Lung Cancer | Urothelial Cancer | Renal Cell Carcinoma | Hepatocellular Carcinoma | Melanoma	DRUG: INCB086550	ALL	ADULT, OLDER_ADULT	PHASE 2	16	INTERVENTIONAL	02/09/2021	26/03/2024	26/03/2024
Nivolumab	Preoperative Immune Checkpoint Inhibitor for Patients With Primary Untreated or Recurrent/Metastatic SCCHN	YES	Head and Neck Squamous Cell Carcinoma | Head and Neck Cancer | Head and Neck Cancer Metastatic	DRUG: Nivolumab 480mg and surgical resection	ALL	ADULT, OLDER_ADULT	PHASE 2	26	INTERVENTIONAL	08/07/2019	17/10/2023	17/10/2023
Bintrafusp alfa	A Phase II Study of Bintrafusp Alfa (M7824) in Checkpoint Inhibitor Naive and Refractory Subjects With Urothelial Carcinoma	YES	Urothelial Cancer	DRUG: Bintrafusp alfa (M7824)	ALL	ADULT, OLDER_ADULT	PHASE 2	2	INTERVENTIONAL	26/10/2020	19/01/2021	13/10/2021
Anti-Nectin-4 ab ADC	A Study of Enfortumab Vedotin for Patients With Locally Advanced or Metastatic Urothelial Bladder Cancer	YES	Carcinoma, Transitional Cell | Urinary Bladder Neoplasms | Urologic Neoplasms | Renal Pelvis Neoplasms | Urothelial Cancer | Ureteral Neoplasms | Urethral Neoplasms	DRUG: Enfortumab vedotin	ALL	ADULT, OLDER_ADULT	PHASE 2	219	INTERVENTIONAL	08/10/2017	27/10/2020	28/07/2023
Bintrafusp Alfa	Phase I/II Trial Investigating the Safety, Tolerability, Pharmacokinetics, Immune and Clinical Activity of SX-682 in Combination With Bintrafusp Alfa (M7824 or TGF-beta "Trap"/PD-L1) With CV301 TRICOM in Advanced Solid Tumors (STAT)	YES	Metastatic Cancer | Solid Tumors	DRUG: SX-682 | DRUG: M7824 | BIOLOGICAL: MVA-BN-CV301 | BIOLOGICAL: recombinant fowlpox viral (FPV)-CV301	ALL	ADULT, OLDER_ADULT	PHASE 1 | PHASE 2	12	INTERVENTIONAL	24/11/2020	07/02/2023	19/05/2023
Pembrolizumab	Lenvatinib Plus Pembrolizumab In Patients With Immune Checkpoint Inhibitor Naive Metastatic Uveal Melanoma	YES	Melanoma, Uveal	DRUG: Pembrolizumab | DRUG: Lenvatinib	ALL	ADULT, OLDER_ADULT	PHASE 2	6	INTERVENTIONAL	02/08/2022	03/12/2024	11/08/2025
Cancer vaccine	OSE2101 Versus Chemotherapy in HLA-A2 Positive Patients With Advanced NSCLC After Immune Checkpoint Inhibitor Failure	YES	Non-Small Cell Lung Cancer	BIOLOGICAL: OSE2101 | DRUG: Docetaxel | DRUG: Pemetrexed	ALL	ADULT, OLDER_ADULT	PHASE 3	219	INTERVENTIONAL	12/02/2016	15/01/2021	15/01/2021
No detailed AE (not specified), ICI in combination with immune modulator (idelalisib)	Pembrolizumab + Idelalisib for Lung Cancer Study	YES	Non-Small Cell Lung Cancer | Metastasis | Recurrence	DRUG: Pembrolizumab | DRUG: Idelalisib	ALL	ADULT, OLDER_ADULT	PHASE 1 | PHASE 2	4	INTERVENTIONAL	26/09/2017	15/08/2020	25/08/2021
Nivolumab	Cytokine Microdialysis for Real-Time Immune Monitoring in Glioblastoma Patients Undergoing Checkpoint Blockade	YES	Glioblastoma	DRUG: Nivolumab | DRUG: BMS-986016	ALL	ADULT, OLDER_ADULT	PHASE 1	21	INTERVENTIONAL	24/09/2018	21/04/2023	27/06/2023
Bintrafusp alfa	Immune Checkpoint Inhibitor M7824 and the Immunocytokine M9241 in Combination With Stereotactic Body Radiation Therapy (SBRT) in Adults With Advanced Pancreatic Cancer	YES	Histologically or Cytologically Confirmed Pancreatic Cancer | Unresectable or Borderline Resectable Pancreatic Cancer | Pancreatic Neoplasms | Pancreatic Cancer | Metastatic Pancreatic Cancer	DRUG: M7824 | DRUG: M9241 | RADIATION: SBRT	ALL	ADULT, OLDER_ADULT	PHASE 1 | PHASE 2	3	INTERVENTIONAL	15/06/2021	18/01/2022	03/02/2022
No detailed AE (not specified)	Effect of Pembrolizumab (Keytruda®) on Biomarkers in Early Breast Cancer	YES	Breast Cancer | Triple Negative Breast Cancer | Hormone Receptor Negative Neoplasm	DRUG: Pembrolizumab	ALL	ADULT, OLDER_ADULT	EARLY_PHASE 1	54	INTERVENTIONAL	18/01/2018	31/03/2020	31/03/2020
Bintrafusp alfa	Combination Immunotherapy in Biochemically Recurrent Prostate Cancer	YES	Prostate Cancer	BIOLOGICAL: Antigen direct immunotherapy: PROSTVAC-V | BIOLOGICAL: Antigen direct immunotherapy: PROSTVAC-F | DRUG: MSB0011359C (M7824) | BIOLOGICAL: Antigen direct immunotherapy: CV301 | DIAGNOSTIC_TEST: CT scan of chest | DIAGNOSTIC_TEST: CT scan of abdomen/pelvis | DIAGNOSTIC_TEST: MRI | DIAGNOSTIC_TEST: PSMA | DIAGNOSTIC_TEST: Bone scan	MALE	ADULT, OLDER_ADULT	PHASE 2	40	INTERVENTIONAL	20/03/2018	04/06/2024	04/06/2024
Atezolizumab	Carboplatin, Etoposide, and Atezolizumab With or Without Trilaciclib (G1T28), a CDK4/6 Inhibitor, in Extensive-Stage SCLC	YES	Small Cell Lung Cancer	DRUG: Trilaciclib | DRUG: Placebo | DRUG: Carboplatin | DRUG: Etoposide | DRUG: Atezolizumab	ALL	ADULT, OLDER_ADULT	PHASE 2	107	INTERVENTIONAL	29/06/2017	17/08/2018	29/10/2020
Not approved anti-PDL1 Ab	PROCLAIM: CX-072-002: Study of PD-L1 Probody Therapeutic CX-072 in Combination With Other Anticancer Therapy in Adults With Solid Tumors	YES	Solid Tumor | Unresectable or Metastatic Melanoma	DRUG: CX-072 | DRUG: Ipilimumab	ALL	ADULT, OLDER_ADULT	PHASE 2	3	INTERVENTIONAL	20/11/2019	21/05/2020	21/05/2020
Pembrolizumab	LUMINOS-101: Lerapolturev (PVSRIPO) and Pembrolizumab in Patients With Recurrent Glioblastoma	YES	Glioblastoma | Recurrent Glioblastoma | Supratentorial Glioblastoma | Brain Tumor	BIOLOGICAL: lerapolturev | BIOLOGICAL: pembrolizumab	ALL	ADULT, OLDER_ADULT	PHASE 2	25	INTERVENTIONAL	21/10/2020	05/06/2024	21/06/2024
Nivolumab	Rational EpigenetiC Immunotherapy for SEcond Line Therapy in Patients With NSCLC: PRECISE Trial	YES	Lung Cancer | Non-Small Cell Lung Cancer	DRUG: Nivolumab | DRUG: oral decitabine | DRUG: Tetrahydrouridine	ALL	ADULT, OLDER_ADULT	PHASE 2	13	INTERVENTIONAL	06/06/2017	02/07/2019	19/05/2024
Pembrolizumab	Pembrolizumab in Combination With Olaparib in Advanced BRCA-mutated or HDR-defect Breast Cancer	YES	Breast Cancer	DRUG: Pembrolizumab | DRUG: Olaparib	ALL	ADULT, OLDER_ADULT	PHASE 2	14	INTERVENTIONAL	10/09/2017	08/10/2024	21/11/2025
Nivolumab Plus Ipilimumab	NIMBUS: Nivolumab Plus Ipilimumab in Metastatic Hypermutated HER2-negative Breast Cancer	YES	Breast Cancer	DRUG: Nivolumab | DRUG: Ipilimumab	ALL	ADULT, OLDER_ADULT	PHASE 2	30	INTERVENTIONAL	08/03/2019	30/09/2021	28/02/2027
Approved PD-1 inhibitor (Retifanlimab)	Anti-GITR/Anti-PD1/Stereotactic Radiosurgery, in Recurrent Glioblastoma	YES	Glioblastoma	DRUG: INCMGA00012 | DRUG: INCAGN01876 | DRUG: SRS | PROCEDURE: Brain surgery	ALL	ADULT, OLDER_ADULT	PHASE 2	39	INTERVENTIONAL	23/06/2020	08/09/2022	30/04/2029
Not approved dendritic cell-based treatment	Polarized Dendritic Cell (aDC1) Based Treatment, Interferon Alpha-2, Rintatolimod, and Celecoxib for the Treatment of HLA-A2 + Refractory Melanoma	YES	HLA-A2-Positive Cells Present | Refractory Melanoma	BIOLOGICAL: Alpha-type-1 Polarized Dendritic Cells | DRUG: Celecoxib | DRUG: PD-1 Ligand Inhibitor | DRUG: PD-1 Inhibitor | BIOLOGICAL: Recombinant Interferon Alfa-2b | DRUG: Rintatolimod	ALL	ADULT, OLDER_ADULT	PHASE 2	1	INTERVENTIONAL	13/11/2024	03/04/2025	19/06/2025
No AE reported	A Study of Duvelisib in Combination With Pembrolizumab in Head and Neck Cancer	YES	Head and Neck Squamous Cell Carcinoma	DRUG: Duvelisib | BIOLOGICAL: Pembrolizumab	ALL	ADULT, OLDER_ADULT	PHASE 1 | PHASE 2	2	INTERVENTIONAL	01/06/2020	10/12/2020	10/12/2020
Pembrolizumab	Pembro and HER2Bi-Armed Activated T Cells in Treating Patients With Metastatic Castration Resistant Prostate Cancer	YES	Castration Levels of Testosterone | Castration-Resistant Prostate Carcinoma | Prostate Carcinoma Metastatic in the Bone | PSA Progression | Stage IV Prostate Adenocarcinoma AJCC v7	BIOLOGICAL: HER2Bi-Armed Activated T Cells | OTHER: Laboratory Biomarker Analysis | BIOLOGICAL: Pembrolizumab	ALL	ADULT, OLDER_ADULT	PHASE 2	15	INTERVENTIONAL	07/06/2018	20/01/2021	07/11/2022
Pembrolizumab	A SU2C Catalyst® Trial of a PD1 Inhibitor With or Without a Vitamin D Analog for the Maintenance of Pancreatic Cancer	YES	Pancreatic Cancer | Pancreas Adenocarcinoma | Advanced Pancreatic Cancer | Metastatic Pancreatic Cancer | Metastatic Pancreatic Adenocarcinoma	DRUG: Pembrolizumab | DRUG: paricalcitol | DRUG: placebo	ALL	ADULT, OLDER_ADULT	PHASE 2	24	INTERVENTIONAL	27/12/2017	29/06/2020	10/07/2020
Pembrolizumab	A Study of Pembrolizumab With Standard Treatment in Patients With Recurrent Platinum-resistant Ovarian Cancer	YES	Ovarian Cancer	DRUG: Pembrolizumab | DRUG: Gemcitabine | DRUG: Cisplatin	FEMALE	ADULT, OLDER_ADULT	PHASE 2	21	INTERVENTIONAL	08/02/2016	17/05/2019	07/03/2022
Not approved anti-PD-L1/CTLA-4 bispecific antibody	A Study of KN046 in Patients With Thymic Carcinoma Who Failed Immune Checkpoint Inhibitors	YES	Thymic Carcinoma	DRUG: KN046	ALL	ADULT, OLDER_ADULT	PHASE 2	4	INTERVENTIONAL	13/12/2021	05/05/2023	05/05/2023
Not approved anti-PD-1 targeting IL-15 fusion protein	Study to Test the Safety and Tolerability of PF-07209960 in Advanced or Metastatic Solid Tumors	YES	Non-Small-cell Lung Cancer | Squamous Cell Carcinoma of the Head and Neck | Renal Cell Carcinoma | Urothelial Carcinoma | Colorectal Carcinoma | Ovarian Carcinoma	BIOLOGICAL: PF-07209960	ALL	ADULT, OLDER_ADULT	PHASE 1	37	INTERVENTIONAL	16/12/2020	03/05/2023	26/05/2023
Nivolumab and Ipilimumab	A Study of Immunotherapy Drugs Nivolumab and Ipilimumab in Patients w/Resectable Malignant Peritoneal Mesothelioma	YES	Mesothelioma | Peritoneal Mesothelioma	DRUG: Nivolumab | DRUG: Ipilimumab	ALL	ADULT, OLDER_ADULT	PHASE 2	2	INTERVENTIONAL	01/12/2021	13/04/2023	13/04/2023
Meet, pembrolizumab (no endocrine AE)	Galinpepimut-S in Combination With Pembrolizumab in Patients With Selected Advanced Cancers	YES	Acute Myelogenous Leukemia | Ovarian Cancer | Colorectal Cancer | Triple-negative Breast Cancer | Small-cell Lung Cancer	BIOLOGICAL: galinpepimut-S | BIOLOGICAL: pembrolizumab | OTHER: Montanide | BIOLOGICAL: GM-CSF	ALL	ADULT, OLDER_ADULT	PHASE 1 | PHASE 2	26	INTERVENTIONAL	30/09/2019	05/07/2022	27/10/2022
Meet, pembrolizumab (no endocrine AE)	Preoperative Anti-PD-1 Antibody Combined With Chemoradiotherapy for Locally Advanced Squamous Cell Carcinoma of Esophagus	YES	Esophageal Squamous Cell Carcinoma	DRUG: Pembrolizumab	ALL	ADULT, OLDER_ADULT	PHASE 1	20	INTERVENTIONAL	21/01/2019	17/03/2020	17/06/2020
Anti-NKG2A ab	Assessment of Efficacy and Safety of Monalizumab Plus Cetuximab Compared to Placebo Plus Cetuximab in Recurrent or Metastatic Head and Neck Cancer	YES	Squamous Cell Carcinoma of the Head and Neck	DRUG: Monalizumab | DRUG: Cetuximab | OTHER: Placebo	ALL	ADULT, OLDER_ADULT	PHASE 3	370	INTERVENTIONAL	02/10/2020	11/05/2022	24/09/2026
Atezolizumab	Derazantinib and Atezolizumab in Patients With Urothelial Cancer	YES	Urothelial Carcinoma	DRUG: Derazantinib 300 mg once daily monotherapy | DRUG: Derazantinib 200 mg once daily + atezolizumab 1200 mg | DRUG: Derazantinib 300 mg once daily + atezolizumab 1200 mg | DRUG: Derazantinib 200 mg twice daily + atezolizumab 1200 mg | DRUG: Derazantinib 300 mg once daily monotherapy (QD) | DRUG: Derazantinib 300 mg once daily + atezolizumab 1200 mg | DRUG: Derazantinib 200 mg twice daily monotherapy	ALL	ADULT, OLDER_ADULT	PHASE 1 | PHASE 2	95	INTERVENTIONAL	02/08/2019	04/10/2022	04/10/2022
Nivolumab	Dabrafenib and Trametinib Followed by Ipilimumab and Nivolumab or Ipilimumab and Nivolumab Followed by Dabrafenib and Trametinib in Treating Patients With Stage III-IV BRAFV600 Melanoma	YES	Clinical Stage III Cutaneous Melanoma AJCC v8 | Clinical Stage IV Cutaneous Melanoma AJCC v8 | Metastatic Melanoma | Recurrent Melanoma | Unresectable Melanoma	PROCEDURE: Biospecimen Collection | PROCEDURE: Computed Tomography | DRUG: Dabrafenib Mesylate | PROCEDURE: Echocardiography Test | BIOLOGICAL: Ipilimumab | PROCEDURE: Multigated Acquisition Scan | BIOLOGICAL: Nivolumab | OTHER: Quality-of-Life Assessment | DRUG: Trametinib Dimethyl Sulfoxide	ALL	ADULT, OLDER_ADULT	PHASE 3	267	INTERVENTIONAL	08/09/2015	30/09/2023	19/12/2026
Nivolumab	Blinatumomab and Nivolumab With or Without Ipilimumab in Treating Patients With Poor-Risk Relapsed or Refractory CD19 + Precursor B-Lymphoblastic Leukemia	YES	Recurrent B Acute Lymphoblastic Leukemia | Recurrent Mixed Phenotype Acute Leukemia | Refractory B Acute Lymphoblastic Leukemia | Refractory Mixed Phenotype Acute Leukemia	PROCEDURE: Biospecimen Collection | BIOLOGICAL: Blinatumomab | PROCEDURE: Bone Marrow Aspiration and Biopsy | BIOLOGICAL: Ipilimumab | BIOLOGICAL: Nivolumab	ALL	CHILD, ADULT, OLDER_ADULT	PHASE 1	28	INTERVENTIONAL	25/10/2017	22/05/2023	18/07/2026
Pembrolizumab	Regorafenib Plus Pembrolizumab in Patients With Advanced or Spreading Liver Cancer Who Have Been Previously Treated With PD-1/PD-L1 Immune Checkpoint Inhibitors	YES	Hepatocellular Carcinoma	DRUG: Pembrolizumab | DRUG: Regorafenib (Stivarga, BAY73-4506)	ALL	ADULT, OLDER_ADULT	PHASE 2	95	INTERVENTIONAL	03/02/2021	05/05/2022	23/04/2024
Approved PD-1 inhibitor	Study of Niraparib, TSR-022, Bevacizumab, and Platinum-Based Doublet Chemotherapy in Combination With TSR-042	YES	Neoplasms | Metastatic Cancer | Advanced Cancer | Solid Tumor | Non-Small Cell Lung Cancer Metastatic | Non-Small Cell Lung Cancer Stage IIIB | Non-Small Cell Lung Cancer	DRUG: Niraparib | DRUG: TSR-042 | DRUG: Carboplatin-Paclitaxel | DRUG: Bevacizumab | DRUG: TSR-022 | DRUG: Carboplatin-Pemetrexed | DRUG: Carboplatin-Nab-Paclitaxel	ALL	ADULT, OLDER_ADULT	PHASE 1	60	INTERVENTIONAL	12/10/2017	26/02/2020	11/12/2024
Chemotherapy Lurbinectedin	Lurbinectedin Monotherapy in Participants With Advanced or Metastatic Solid Tumors	YES	Advanced Solid Tumor | Metastatic Solid Tumor | Urothelial Cancer | Poorly Differentiated Neuroendocrine Carcinomas | Homologous Recombination Deficient-Positive Malignancies Agnostic	DRUG: Lurbinectedin	ALL	ADULT, OLDER_ADULT	PHASE 2	47	INTERVENTIONAL	03/03/2022	20/12/2023	20/12/2023
ICI, RT	Hypofractionated Radiation Therapy to Improve Immunotherapy Response in Non-Small Cell Lung Cancer	YES	Non-Small Cell Lung Cancer Metastatic	RADIATION: Radiation | DRUG: Immuno-Therapeutic Agent	ALL	ADULT, OLDER_ADULT	NA	35	INTERVENTIONAL	23/01/2017	30/06/2022	30/06/2022
ICI + TKI	Serial Ultrasound in Metastatic Renal Cell Carcinoma (mRCC)	YES	Kidney Cancer | Renal Cell Carcinoma | Metastatic Renal Cell Carcinoma	DIAGNOSTIC_TEST: Doppler Ultrasound | DEVICE: SIEMENS S3000 and Verasonics Vantage 256 | DRUG: Standard-of-care Vascular Endothelial Growth Factor Receptor 2 (VEGFR2) tyrosine kinase inhibitor (TKI) plus immune checkpoint inhibitor (ICI) | DRUG: Standard-of-care non-immune checkpoint inhibitor (ICI) such as single-agent VEGFR2 TKI	ALL	ADULT, OLDER_ADULT	NA	22	INTERVENTIONAL	05/12/2020	30/11/2023	30/11/2023
ICI, not approved STING agonist	Study of PULSAR-ICI ± IMSA101 in Patients With Oligometastatic NSCLC and RCC	YES	Oligometastatic Disease	DRUG: IMSA101 | DRUG: Immune checkpoint inhibitor | RADIATION: PULSAR	ALL	ADULT, OLDER_ADULT	PHASE 2	6	INTERVENTIONAL	28/06/2023	16/09/2024	16/09/2024
Hormonal therapy	Fulvestrant in Treating Patients With Recurrent, Persistent, or Metastatic Endometrial Cancer	YES	Recurrent Uterine Corpus Carcinoma | Stage III Uterine Corpus Cancer AJCC v7 | Stage IV Uterine Corpus Cancer AJCC v7	DRUG: Fulvestrant	FEMALE	ADULT, OLDER_ADULT	PHASE 2	67	INTERVENTIONAL	30/08/2004	17/03/2008	
ICI, not approved STING agonist	Safety and Efficacy Study of IMSA101 in Refractory Malignancies	YES	Solid Tumor, Adult	DRUG: IMSA101 | DRUG: Immune checkpoint inhibitor (ICI) | DRUG: Immuno-oncology (IO) therapy	ALL	ADULT, OLDER_ADULT	PHASE 1 | PHASE 2	40	INTERVENTIONAL	23/09/2019	15/09/2023	15/09/2023
ICI	Trying to Find the Correct Length of Treatment With Immune Checkpoint Therapy	YES	Locally Advanced Bladder Urothelial Carcinoma | Locally Advanced Renal Pelvis Urothelial Carcinoma | Locally Advanced Ureter Urothelial Carcinoma | Locally Advanced Urethral Urothelial Carcinoma | Locally Advanced Urothelial Carcinoma | Metastatic Bladder Urothelial Carcinoma | Metastatic Renal Pelvis Urothelial Carcinoma | Metastatic Ureter Urothelial Carcinoma | Metastatic Urethral Urothelial Carcinoma | Metastatic Urothelial Carcinoma	DRUG: Pembrolizumab | DRUG: Nivolumab | DRUG: Atezolizumab | DRUG: Durvalumab | DRUG: Avelumab	ALL	ADULT, OLDER_ADULT	PHASE 3	3	INTERVENTIONAL	27/05/2022	01/06/2024	2030/09
Targeted therapy + hormonal therapy	Alisertib With or Without Fulvestrant in Treating Patients With Locally Advanced or Metastatic, Endocrine-Resistant Breast Cancer	YES	Estrogen Receptor Status | HER2/Neu Negative | Invasive Breast Carcinoma | Postmenopausal | Stage III Breast Cancer | Stage IIIA Breast Cancer | Stage IIIB Breast Cancer | Stage IIIC Breast Cancer | Stage IV Breast Cancer	DRUG: Alisertib | DRUG: Fulvestrant | OTHER: Laboratory Biomarker Analysis	FEMALE	ADULT, OLDER_ADULT	PHASE 2	96	INTERVENTIONAL	06/07/2017	10/01/2022	14/02/2025
Hormonal therapy	Aromatase Inhibitor Therapy With or Without Fulvestrant for the Treatment of HR Positive Metastatic Breast Cancer With an ERS1 Activating Mutation, the INTERACT Study	YES	Anatomic Stage IV Breast Cancer AJCC v8 | Metastatic Breast Carcinoma | Prognostic Stage IV Breast Cancer AJCC v8	DRUG: Abemaciclib | DRUG: Anastrozole | DRUG: Fulvestrant | DRUG: Letrozole | DRUG: Palbociclib | DRUG: Ribociclib	ALL	ADULT, OLDER_ADULT	PHASE 2	4	INTERVENTIONAL	31/05/2019	22/09/2023	22/09/2023
Hormonal therapy	Fulvestrant in Hormone Refractory Prostate Cancer	YES	Prostatic Neoplasms | Prostate Cancer	DRUG: Fulvestrant	MALE	ADULT, OLDER_ADULT	PHASE 2	10	INTERVENTIONAL	2006/09	2009/09	2009/12
Hormonal therapy (aromatase inhibitors)	Anastrozole and Letrozole After Surgery for the Treatment of Stage I-III Breast Cancer	YES	Anatomic Stage I Breast Cancer AJCC v8 | Anatomic Stage IA Breast Cancer AJCC v8 | Anatomic Stage IB Breast Cancer AJCC v8 | Anatomic Stage II Breast Cancer AJCC v8 | Anatomic Stage IIA Breast Cancer AJCC v8 | Anatomic Stage IIB Breast Cancer AJCC v8 | Anatomic Stage III Breast Cancer AJCC v8 | Anatomic Stage IIIA Breast Cancer AJCC v8 | Anatomic Stage IIIB Breast Cancer AJCC v8 | Anatomic Stage IIIC Breast Cancer AJCC v8 | Early-Stage Breast Carcinoma | Invasive Breast Carcinoma | Prognostic Stage I Breast Cancer AJCC v8 | Prognostic Stage IA Breast Cancer AJCC v8 | Prognostic Stage IB Breast Cancer AJCC v8 | Prognostic Stage II Breast Cancer AJCC v8 | Prognostic Stage IIA Breast Cancer AJCC v8 | Prognostic Stage IIB Breast Cancer AJCC v8 | Prognostic Stage III Breast Cancer AJCC v8 | Prognostic Stage IIIA Breast Cancer AJCC v8 | Prognostic Stage IIIB Breast Cancer AJCC v8 | Prognostic Stage IIIC Breast Cancer AJCC v8	DRUG: Anastrozole | DRUG: Letrozole	FEMALE	ADULT, OLDER_ADULT	PHASE 2	161	INTERVENTIONAL	28/04/2020	12/12/2022	12/12/2022
Targeted therapy TKI	Pazopanib Hydrochloride With or Without Bicalutamide in Treating Patients With Prostate Cancer That Did Not Respond to Hormone Therapy	YES	Hormone-Resistant Prostate Cancer | Recurrent Prostate Carcinoma	DRUG: Bicalutamide | OTHER: Laboratory Biomarker Analysis | DRUG: Pazopanib Hydrochloride | OTHER: Pharmacological Study	MALE	ADULT, OLDER_ADULT	PHASE 2	23	INTERVENTIONAL	2007/09	2015/08	2015/09
Hormonal therapy + TKI	Fulvestrant With or Without Lapatinib in Treating Postmenopausal Women With Stage III or Stage IV Breast Cancer That is Hormone Receptor-Positive	YES	Estrogen Receptor Positive | HER2 Positive Breast Carcinoma | HER2/Neu Negative | Progesterone Receptor Positive | Recurrent Breast Carcinoma | Stage IIIB Breast Cancer AJCC v7 | Stage IIIC Breast Cancer AJCC v7 | Stage IV Breast Cancer AJCC v6 and v7	DRUG: Fulvestrant | OTHER: Laboratory Biomarker Analysis | DRUG: Lapatinib Ditosylate | OTHER: Placebo Administration	FEMALE	ADULT, OLDER_ADULT	PHASE 3	295	INTERVENTIONAL	15/09/2006	01/07/2014	02/07/2014
TKI + hormonal therapy (aromatase inhibitors)	Anastrozole and ZD1839 Compared With Fulvestrant and ZD1839 in Postmenopausal Women With Metastatic Breast Cancer	YES	Estrogen Receptor-positive Breast Cancer | Progesterone Receptor-positive Breast Cancer | Recurrent Breast Cancer | Stage IV Breast Cancer	DRUG: anastrozole | DRUG: gefitinib | DRUG: fulvestrant | OTHER: laboratory biomarker analysis	FEMALE	ADULT, OLDER_ADULT	PHASE 2	148	INTERVENTIONAL	2003/09	2012/06	2012/06
ICI, RT	Immune Checkpoint Inhibitor and MR-guided SBRT for Limited Progressive Metastatic Carcinoma	YES	Metastatic Carcinoma	RADIATION: Radiation Therapy	ALL	ADULT, OLDER_ADULT	NA	12	INTERVENTIONAL	08/04/2020	16/06/2023	04/10/2023
Proteasome inhibitor + hormonal therapy	Fulvestrant With or Without Bortezomib in Patients With Inoperable Locally Advanced or Metastatic Estrogen Receptor Positive Breast Cancer	YES	Metastatic Breast Carcinoma | Stage IIIB Breast Cancer AJCC v7 | Stage IIIC Breast Cancer AJCC v7 | Stage IV Breast Cancer AJCC v6 and v7	DRUG: Bortezomib | DRUG: Fulvestrant | OTHER: Laboratory Biomarker Analysis	FEMALE	ADULT, OLDER_ADULT	PHASE 2	118	INTERVENTIONAL	26/05/2010	30/07/2015	08/01/2021
Androgen receptor inhibitor + Akt inhibitor	Bicalutamide With or Without Akt Inhibitor MK2206 in Treating Patients With Previously Treated Prostate Cancer	YES	Recurrent Prostate Carcinoma | Stage I Prostate Cancer AJCC v7 | Stage IIA Prostate Cancer AJCC v7 | Stage IIB Prostate Cancer AJCC v7 | Stage III Prostate Cancer AJCC v7	DRUG: Akt Inhibitor MK2206 | DRUG: Bicalutamide | OTHER: Clinical Observation | OTHER: Laboratory Biomarker Analysis	MALE	ADULT, OLDER_ADULT	PHASE 2	108	INTERVENTIONAL	17/03/2011	17/07/2018	26/05/2026
Not approved FTI + hormonal therapy	Tipifarnib and Fulvestrant in Hormone Receptor-Positive Metastatic Breast Cancer	YES	Estrogen Receptor-positive Breast Cancer | Recurrent Breast Cancer | Stage IIIB Breast Cancer | Stage IIIC Breast Cancer | Stage IV Breast Cancer	DRUG: fulvestrant | DRUG: tipifarnib	FEMALE	ADULT, OLDER_ADULT	PHASE 2	33	INTERVENTIONAL	2004/03	2008/07	2008/09
TKI + hormonal therapy	Neo-adjuvant Therapy With Anastrozole Plus Pazopanib in Stage II and III ER + Breast Cancer	YES	Estrogen Receptor-positive Breast Cancer | Human Epidermal Growth Factor 2 Negative Carcinoma of Breast | Male Breast Cancer | Recurrent Breast Cancer | Stage II Breast Cancer | Stage IIIA Breast Cancer | Stage IIIB Breast Cancer	DRUG: anastrozole | DRUG: pazopanib hydrochloride | PROCEDURE: therapeutic conventional surgery	ALL	ADULT, OLDER_ADULT	PHASE 2	2	INTERVENTIONAL	13/07/2011	12/04/2012	12/04/2012
Akt inhibitor, TKI	Akt Inhibitor MK-2206 and Anastrozole With or Without Goserelin Acetate in Treating Patients With Stage II-III Breast Cancer	YES	Estrogen Receptor Positive | HER2/Neu Negative | Recurrent Breast Carcinoma | Stage IIA Breast Cancer | Stage IIB Breast Cancer | Stage IIIA Breast Cancer | Stage IIIB Breast Cancer | Stage IIIC Breast Cancer	DRUG: Akt Inhibitor MK2206 | DRUG: Anastrozole | DRUG: Goserelin Acetate | OTHER: Laboratory Biomarker Analysis | PROCEDURE: Neoadjuvant Therapy | OTHER: Pharmacological Study | PROCEDURE: Therapeutic Conventional Surgery	FEMALE	ADULT, OLDER_ADULT	PHASE 2	16	INTERVENTIONAL	2013/01	2015/05	05/05/2015
Radiation therapy, targeted therapy, and hormone therapy	Radiation Therapy, Palbociclib, and Hormone Therapy in Treating Breast Cancer Patients With Bone Metastasis	YES	Anatomic Stage IV Breast Cancer AJCC v8 | Estrogen Receptor Positive | HER2/Neu Negative | Metastatic Breast Carcinoma | Metastatic Malignant Neoplasm in the Bone | Progesterone Receptor Positive | Prognostic Stage IV Breast Cancer AJCC v8	DRUG: Anastrozole | DRUG: Exemestane | DRUG: Fulvestrant | DRUG: Letrozole | DRUG: Palbociclib | RADIATION: Radiation Therapy | DRUG: Tamoxifen	ALL	ADULT, OLDER_ADULT	PHASE 2	36	INTERVENTIONAL	08/02/2019	13/10/2022	13/10/2022
mTOR inhibitor	Phase II Study of Everolimus Beyond Progression	YES	Estrogen Receptor-positive Breast Cancer | HER2-negative Breast Cancer | Progesterone Receptor-positive Breast Cancer | Recurrent Breast Cancer | Stage IIIA Breast Cancer | Stage IIIB Breast Cancer | Stage IIIC Breast Cancer | Stage IV Breast Cancer	DRUG: everolimus | DRUG: anastrozole | DRUG: letrozole | DRUG: tamoxifen citrate | DRUG: fulvestrant | DRUG: megestrol acetate	FEMALE	ADULT, OLDER_ADULT	PHASE 2	3	INTERVENTIONAL	25/11/2014	25/01/2021	25/01/2021
ICI	PET/CT Quantitative Assessment of Myocardial Blood Flow Changes in Oncologic Patients Receiving Checkpoint Inhibitor Therapy	YES	Cancer	DIAGNOSTIC_TEST: PET/CT | DRUG: radioactive drug (tracer)	ALL	ADULT, OLDER_ADULT	PHASE 1 | PHASE 2	6	INTERVENTIONAL	14/08/2020	19/05/2022	19/05/2022
Hormone therapy, chemotherapy	Tamoxifen Citrate, Letrozole, Anastrozole, or Exemestane With or Without Chemotherapy in Treating Patients With Invasive RxPONDER Breast Cancer	YES	Breast Ductal Carcinoma In Situ | Invasive Breast Carcinoma | Multicentric Breast Carcinoma | Multifocal Breast Carcinoma | Synchronous Bilateral Breast Carcinoma	DRUG: Anastrozole | DRUG: Exemestane | OTHER: Laboratory Biomarker Analysis | DRUG: Letrozole | OTHER: Quality-of-Life Assessment | DRUG: Systemic Chemotherapy | DRUG: Tamoxifen Citrate	FEMALE	ADULT, OLDER_ADULT	PHASE 3	5018	INTERVENTIONAL	28/02/2011	01/02/2023	08/01/2027
Hormone therapy, chemotherapy	Hormone Therapy With or Without Combination Chemotherapy in Treating Women Who Have Undergone Surgery for Node-Negative Breast Cancer (The TAILORx Trial)	YES	Breast Adenocarcinoma | Hormone Receptor Positive | Stage IA Breast Cancer AJCC v7 | Stage IB Breast Cancer AJCC v7 | Stage IIA Breast Cancer AJCC v6 and v7 | Stage IIB Breast Cancer AJCC v6 and v7 | Stage IIIB Breast Cancer AJCC v7	DRUG: Anastrozole | DRUG: Exemestane | OTHER: Laboratory Biomarker Analysis | DRUG: Letrozole | OTHER: Quality-of-Life Assessment | RADIATION: Radiation Therapy | DRUG: Tamoxifen Citrate	FEMALE	ADULT, OLDER_ADULT	PHASE 3	10273	INTERVENTIONAL	07/04/2006	02/03/2018	30/09/2030
Not approved cancer vaccine	A Phase II Study of PROSTVAC-V (Vaccinia)/TRICOM and PROSTVAC-F (Fowlpox)/TRICOM With GM-CSF in Patients With PSA Progression After Local Therapy for Prostate Cancer	YES	Recurrent Prostate Carcinoma | Stage I Prostate Cancer | Stage IIA Prostate Cancer | Stage IIB Prostate Cancer | Stage III Prostate Cancer	DRUG: Bicalutamide | DRUG: Goserelin Acetate | BIOLOGICAL: Recombinant Fowlpox-PSA(L155)/TRICOM Vaccine | BIOLOGICAL: Recombinant Vaccinia-TRICOM Vaccine | BIOLOGICAL: Sargramostim	MALE	ADULT, OLDER_ADULT	PHASE 2	50	INTERVENTIONAL	2006/02	2010/10	2010/10
Hormone therapy	Tamoxifen Citrate or Z-Endoxifen Hydrochloride in Treating Patients With Locally Advanced or Metastatic, Estrogen Receptor-Positive, HER2-Negative Breast Cancer	YES	Recurrent Breast Carcinoma | Stage III Breast Cancer AJCC v7 | Stage IIIA Breast Cancer AJCC v7 | Stage IIIB Breast Cancer AJCC v7 | Stage IIIC Breast Cancer AJCC v7 | Stage IV Breast Cancer AJCC v6 and v7	DRUG: Endoxifen Hydrochloride | OTHER: Laboratory Biomarker Analysis | OTHER: Pharmacological Study | DRUG: Tamoxifen Citrate	FEMALE	ADULT, OLDER_ADULT	PHASE 2	81	INTERVENTIONAL	28/05/2015	06/11/2020	02/02/2027
TKI and hormonal therapy	Sorafenib and Anastrozole in Treating Postmenopausal Women With Metastatic Breast Cancer	YES	Recurrent Breast Cancer | Stage IV Breast Cancer	DRUG: sorafenib tosylate | DRUG: anastrozole	FEMALE	ADULT, OLDER_ADULT	PHASE 1 | PHASE 2	35	INTERVENTIONAL	2005/06	2009/07	2013/01
Androgen deprivation therapy + HDAC (histone deacetylase) inhibitor	Androgen Deprivation Therapy and Vorinostat Followed by Radical Prostatectomy in Treating Patients With Localized Prostate Cancer	YES	Prostate Adenocarcinoma | Stage I Prostate Cancer | Stage IIA Prostate Cancer | Stage IIB Prostate Cancer | Stage III Prostate Cancer	DRUG: Bicalutamide | DRUG: Goserelin Acetate | OTHER: Laboratory Biomarker Analysis | DRUG: Leuprolide Acetate | PROCEDURE: Therapeutic Conventional Surgery | DRUG: Vorinostat	MALE	ADULT, OLDER_ADULT	PHASE 2	19	INTERVENTIONAL	2007/11	2010/06	2010/06
Hormone therapy	Onapristone and Anastrozole for the Treatment of Refractory Hormone Receptor Positive Endometrial Cancer	YES	Refractory Endometrial Adenocarcinoma | Refractory Endometrial Carcinoma | Refractory Endometrial Clear Cell Adenocarcinoma | Refractory Endometrial Endometrioid Adenocarcinoma | Refractory Endometrial Mixed Cell Adenocarcinoma | Refractory Endometrial Serous Adenocarcinoma | Refractory Endometrial Undifferentiated Carcinoma	DRUG: Extended-release Onapristone | DRUG: Anastrozole | OTHER: Quality-of-Life Assessment | OTHER: Questionnaire Administration | DIAGNOSTIC_TEST: Estrogen Receptor Positive (Positive Estrogen Receptor; ESR Positive; ESR1 Positive; ER Positive; Estrogen Receptor Alpha Positive) | DIAGNOSTIC_TEST: Progesterone Receptor Positive (PGR Positive; PR Positive)	FEMALE	ADULT, OLDER_ADULT	PHASE 2	14	INTERVENTIONAL	28/01/2021	01/03/2025	17/12/2025
Hormone therapy	Goserelin and Letrozole or Anastrozole in Premenopausal Patients With Stage II-III Estrogen Receptor-Positive Breast Cancer	YES	Estrogen Receptor-positive Breast Cancer | HER2-negative Breast Cancer | Stage II Breast Cancer | Stage IIIA Breast Cancer | Stage IIIB Breast Cancer | Stage IIIC Breast Cancer	DRUG: goserelin acetate | DRUG: letrozole | DRUG: anastrozole | DRUG: chemotherapy | PROCEDURE: Surgery	FEMALE	ADULT, OLDER_ADULT	PHASE 2	8	INTERVENTIONAL	2011/09	2013/05	2013/05
Androgen deprivation therapy + anti-IGF-1R Ab(target therapy)	Bicalutamide and Goserelin or Leuprolide Acetate With or Without Cixutumumab in Treating Patients With Newly Diagnosed Metastatic Prostate Cancer	YES	Prostate Adenocarcinoma | Recurrent Prostate Carcinoma | Stage IV Prostate Cancer	DRUG: Bicalutamide | BIOLOGICAL: Cixutumumab | DRUG: Goserelin Acetate | OTHER: Laboratory Biomarker Analysis | DRUG: Leuprolide Acetate | OTHER: Pharmacological Study	MALE	ADULT, OLDER_ADULT	PHASE 2	211	INTERVENTIONAL	2010/12	2015/06	23/08/2017
Hormone therapy + RT	Hormone Therapy and Intensity-Modulated Radiation Therapy in Treating Patients With Metastatic Prostate Cancer	YES	PSA Level Greater Than Two | Stage IV Prostate Adenocarcinoma AJCC v7	DRUG: Bicalutamide | DRUG: Goserelin Acetate | RADIATION: Intensity-Modulated Radiation Therapy | OTHER: Laboratory Biomarker Analysis | DRUG: Leuprolide Acetate	MALE	ADULT, OLDER_ADULT	PHASE 2	29	INTERVENTIONAL	18/07/2006	16/03/2011	15/02/2027
Hormone therapy	Low-Dose Tamoxifen Citrate in Reducing Breast Cancer Risk in Radiation-Induced Cancer Survivors	YES	Breast Cancer	DRUG: Tamoxifen Citrate | DRUG: Placebo | PROCEDURE: Digital mammography | OTHER: immunohistochemistry staining method | OTHER: pharmacological study | OTHER: laboratory biomarker analysis | GENETIC: protein expression analysis | OTHER: pharmacogenomic studies | OTHER: questionnaire administration | PROCEDURE: Fine needle aspiration | OTHER: Quality of Life Assessment	FEMALE	ADULT, OLDER_ADULT	PHASE 2	84	INTERVENTIONAL	2010/09	11/12/2018	11/12/2028
Fatty acid synthase (FASN) inhibitor, chemo, hormone therapy	TVB-2640 and Trastuzumab With Paclitaxel or Endocrine Therapy for Treatment of HER2 Positive Metastatic Breast Cancer	YES	Advanced Breast Carcinoma | HER2-Positive Breast Carcinoma | Anatomic Stage III Breast Cancer AJCC v8 | Anatomic Stage IV Breast Cancer AJCC v8 | HER2-positive Breast Cancer	DRUG: Anastrozole | DRUG: Denifanstat | DRUG: Exemestane | DRUG: Fulvestrant | PROCEDURE: Echocardiography | DRUG: Letrozole | DRUG: Paclitaxel | BIOLOGICAL: Trastuzumab | PROCEDURE: Computed Tomography | PROCEDURE: Magnetic Resonance Imaging | PROCEDURE: Biospecimen Collection | PROCEDURE: Biopsy	ALL	ADULT, OLDER_ADULT	PHASE 2	17	INTERVENTIONAL	03/08/2017	30/07/2024	13/03/2026
PI3K inhibitor + CDK4/6 inhibitor + hormonal therapy	Testing the Addition of Copanlisib to Usual Treatment (Fulvestrant and Abemaciclib) in Metastatic Breast Cancer	YES	Anatomic Stage IV Breast Cancer AJCC v8 | Metastatic Breast Carcinoma | Metastatic HER2-Negative Breast Carcinoma | Metastatic Hormone Receptor-Positive Breast Carcinoma | Recurrent Breast Carcinoma	DRUG: Abemaciclib | PROCEDURE: Biopsy Procedure | PROCEDURE: Biospecimen Collection | DRUG: Copanlisib Hydrochloride | PROCEDURE: Diagnostic Imaging Testing | PROCEDURE: Echocardiography Test | DRUG: Fulvestrant | PROCEDURE: Multigated Acquisition Scan	ALL	ADULT, OLDER_ADULT	PHASE 1	24	INTERVENTIONAL	17/06/2020	22/06/2023	14/08/2026
Hormone therapy	Vorinostat in Treating Patients With Stage IV Breast Cancer Receiving Hormone Therapy	YES	Male Breast Cancer | Recurrent Breast Cancer | Stage IV Breast Cancer	DRUG: vorinostat | DRUG: anastrozole | DRUG: letrozole | DRUG: exemestane | PROCEDURE: positron emission tomography | RADIATION: F-18 16 alpha-fluoroestradiol | RADIATION: fludeoxyglucose F 18 | OTHER: laboratory biomarker analysis	ALL	ADULT, OLDER_ADULT	NA	15	INTERVENTIONAL	2012/11	2015/03	2020/01
HDAC inhibitor	Vorinostat in Treating Patients With Stage IV Breast Cancer Receiving Aromatase Inhibitor Therapy	YES	Male Breast Cancer | Recurrent Breast Cancer | Stage IV Breast Cancer	DRUG: vorinostat | OTHER: laboratory biomarker analysis | PROCEDURE: biopsy | RADIATION: F-18 16 alpha-fluoroestradiol | PROCEDURE: positron emission tomography | DRUG: anastrozole | DRUG: letrozole | DRUG: exemestane | GENETIC: gene expression analysis	ALL	ADULT, OLDER_ADULT	NA	8	INTERVENTIONAL	2010/11	2012/07	11/08/2016
MEK inhibitor	Trametinib in Treating Patients With Recurrent or Progressive Low-Grade Ovarian Cancer or Peritoneal Cavity Cancer	YES	Borderline Ovarian Serous Tumor | Micropapillary Serous Carcinoma | Ovarian Serous Adenocarcinoma | Primary Peritoneal Serous Adenocarcinoma | Recurrent Ovarian Low Grade Serous Adenocarcinoma | Recurrent Primary Peritoneal Serous Adenocarcinoma	DRUG: Letrozole | DRUG: Paclitaxel | DRUG: Pegylated Liposomal Doxorubicin Hydrochloride | OTHER: Quality-of-Life Assessment | OTHER: Questionnaire Administration | DRUG: Tamoxifen Citrate | DRUG: Topotecan | DRUG: Trametinib Dimethyl Sulfoxide	FEMALE	ADULT, OLDER_ADULT	PHASE 2 | PHASE 3	260	INTERVENTIONAL	11/04/2014	04/04/2019	27/10/2025
TKI and hormonal therapy	Lapatinib and Tamoxifen in Treating Patients With Locally Advanced or Metastatic Breast Cancer That Did Not Respond to Previous Tamoxifen	YES	Estrogen Receptor Positive | Male Breast Carcinoma | Progesterone Receptor Positive | Recurrent Breast Carcinoma | Stage IIIB Breast Cancer | Stage IIIC Breast Cancer | Stage IV Breast Cancer	OTHER: Laboratory Biomarker Analysis | DRUG: Lapatinib Ditosylate | DRUG: Tamoxifen Citrate	ALL	ADULT, OLDER_ADULT	PHASE 2	19	INTERVENTIONAL	2005/05	2011/07	2014/09
mTOR inhibitor + hormonal therapy	Temsirolimus With or Without Megestrol Acetate and Tamoxifen Citrate in Treating Patients With Advanced, Persistent, or Recurrent Endometrial Cancer	YES	Endometrial Carcinoma | Recurrent Uterine Corpus Carcinoma | Stage IIIA Uterine Corpus Cancer | Stage IIIB Uterine Corpus Cancer | Stage IIIC1 Uterine Corpus Cancer | Stage IIIC2 Uterine Corpus Cancer | Stage IVA Uterine Corpus Cancer | Stage IVB Uterine Corpus Cancer	OTHER: Laboratory Biomarker Analysis | DRUG: Megestrol Acetate | DRUG: Tamoxifen Citrate | DRUG: Temsirolimus	FEMALE	CHILD, ADULT, OLDER_ADULT	PHASE 2	73	INTERVENTIONAL	2008/09	2017/02	2017/02
Hormone therapy	Testing an Active Form of Tamoxifen (4-hydroxytamoxifen) Delivered Through Breast Skin to Control Ductal Carcinoma in Situ (DCIS) of the Breast	YES	Ductal Breast Carcinoma In Situ | Estrogen Receptor Positive	DRUG: Afimoxifene | OTHER: Laboratory Biomarker Analysis | OTHER: Placebo | OTHER: Placebo | DRUG: Tamoxifen Citrate	FEMALE	ADULT, OLDER_ADULT	PHASE 2	100	INTERVENTIONAL	31/05/2017	30/03/2021	24/01/2022
Hormone therapy	Suppression of Ovarian Function With Either Tamoxifen or Exemestane Compared With Tamoxifen Alone in Treating Premenopausal Women With Hormone-Responsive Breast Cancer	YES	Estrogen Receptor Positive Breast Cancer | Progesterone Receptor Positive Tumor | Recurrent Breast Carcinoma | Stage IA Breast Cancer | Stage IB Breast Cancer | Stage IIA Breast Cancer | Stage IIB Breast Cancer | Stage IIIA Breast Cancer	DRUG: Exemestane | OTHER: Laboratory Biomarker Analysis | PROCEDURE: Oophorectomy | OTHER: Quality-of-Life Assessment | RADIATION: Radiation Therapy | DRUG: Tamoxifen | DRUG: Triptorelin	FEMALE	ADULT, OLDER_ADULT	PHASE 3	3066	INTERVENTIONAL	17/12/2003	2014/05	06/11/2024
Targeted therapy, hormone therapy	Tamoxifen Citrate or Letrozole With or Without Bevacizumab in Treating Women With Stage IIIB or Stage IV Breast Cancer	YES	Invasive Breast Carcinoma | Recurrent Breast Carcinoma | Stage III Breast Cancer AJCC v6 | Stage IV Breast Cancer AJCC v6 and v7	BIOLOGICAL: Bevacizumab | OTHER: Laboratory Biomarker Analysis | DRUG: Letrozole | OTHER: Questionnaire Administration | DRUG: Tamoxifen Citrate	FEMALE	ADULT, OLDER_ADULT	PHASE 3	394	INTERVENTIONAL	24/09/2008	30/06/2014	01/05/2025
Adverse event data were not collected	Immune-checkpoint Inhibitors and Surrogate Endpoints in Cancer Trials (SURROGATE-ICI)	YES	Cancer Treated With Immune-checkpoint Inhibitors	DRUG: nivolumab monotherapy | DRUG: nivolumab plus ipilimumab	ALL	ADULT, OLDER_ADULT		631	OBSERVATIONAL	2017/01	2020/12	2020/12
Avelumab	A Study of Avelumab With Axitinib Versus Sunitinib In Advanced Renal Cell Cancer (JAVELIN Renal 101)	YES	Renal Cell Cancer	DRUG: Avelumab (MSB0010718C) | DRUG: Axitinib (AG-013736) | DRUG: Sunitinib	ALL	ADULT, OLDER_ADULT	PHASE 3	886	INTERVENTIONAL	23/03/2016	31/08/2023	26/06/2024
Nivolumab	An Investigational Immuno-therapy Trial of Nivolumab, or Nivolumab Plus Ipilimumab, or Nivolumab Plus Platinum-doublet Chemotherapy, Compared to Platinum Doublet Chemotherapy in Patients With Stage IV Non-Small Cell Lung Cancer (NSCLC)	YES	Non-Small Cell Lung Cancer	DRUG: Nivolumab | DRUG: Ipilimumab | DRUG: Carboplatin | DRUG: Cisplatin | DRUG: Gemcitabine | DRUG: Pemetrexed | DRUG: Paclitaxel	ALL	ADULT, OLDER_ADULT	PHASE 3	2747	INTERVENTIONAL	05/08/2015	25/10/2024	25/10/2024
Atezolizumab	A Study of Atezolizumab in Combination With Carboplatin Plus (+) Paclitaxel With or Without Bevacizumab Compared With Carboplatin + Paclitaxel + Bevacizumab in Participants With Stage IV Non-Squamous Non-Small Cell Lung Cancer (NSCLC)	YES	Carcinoma, Non-Small-Cell Lung	DRUG: Atezolizumab (MPDL3280A), an engineered anti-PD-L1 antibody | DRUG: Bevacizumab | DRUG: Carboplatin | DRUG: Paclitaxel	ALL	ADULT, OLDER_ADULT	PHASE 3	1202	INTERVENTIONAL	31/03/2015	13/09/2019	07/12/2020
Pembrolizumab + Epacadostat	Study to Explore the Safety, Tolerability and Efficacy of MK-3475 in Combination With INCB024360 in Participants With Selected Cancers	YES	Microsatellite-instability (MSI) High Colorectal Cancer (CRC) | Endometrial Cancer | Head and Neck Cancer | Hepatocellular Carcinoma (HCC) | Gastric Cancer | Lung Cancer | Lymphoma | Renal Cell Carcinoma (RCC) | Ovarian Cancer | Solid Tumors | UC (Urothelial Cancer) | Melanoma | Bladder Cancer | Triple Negative Breast Cancer (TNBC)	DRUG: MK-3475 | DRUG: INCB024360	ALL	ADULT, OLDER_ADULT	PHASE 1 | PHASE 2	444	INTERVENTIONAL	17/07/2014	26/11/2018	06/11/2020
Adverse event data were not collected	Lung-MAP: Biomarker-Targeted Second-Line Therapy in Treating Patients With Recurrent Stage IV Squamous Cell Lung Cancer	YES	Recurrent Squamous Cell Lung Carcinoma | Stage IV Squamous Cell Lung Carcinoma AJCC v7	DRUG: Docetaxel | BIOLOGICAL: Durvalumab | DRUG: Erlotinib Hydrochloride | DRUG: FGFR Inhibitor AZD4547 | BIOLOGICAL: Ipilimumab | OTHER: Laboratory Biomarker Analysis | BIOLOGICAL: Nivolumab | DRUG: Palbociclib | OTHER: Pharmacological Study | BIOLOGICAL: Rilotumumab | DRUG: Talazoparib | DRUG: Taselisib | BIOLOGICAL: Tremelimumab	ALL	ADULT, OLDER_ADULT		1864	OBSERVATIONAL	08/07/2014	01/04/2022	15/04/2022
Atezolizumab	A Study of Atezolizumab Compared With Docetaxel in Participants With Locally Advanced or Metastatic Non-Small Cell Lung Cancer Who Have Failed Platinum-Containing Therapy	YES	Non-Squamous Non-Small Cell Lung Cancer	DRUG: Atezolizumab | DRUG: Docetaxel	ALL	ADULT, OLDER_ADULT	PHASE 3	1225	INTERVENTIONAL	11/03/2014	07/07/2016	09/01/2019
Pembrolizumab	Study of Two Doses of Pembrolizumab (MK-3475) Versus Docetaxel in Previously Treated Participants With Non-Small Cell Lung Cancer (MK-3475-010/KEYNOTE-010)	YES	Non-Small Cell Lung Cancer (NSCLC)	BIOLOGICAL: Pembrolizumab | DRUG: Docetaxel	ALL	ADULT, OLDER_ADULT	PHASE 2 | PHASE 3	1034	INTERVENTIONAL	09/08/2013	30/09/2015	30/09/2020
Pembrolizumab	Study to Evaluate the Safety and Efficacy of Two Different Dosing Schedules of Pembrolizumab (MK-3475) Compared to Ipilimumab in Participants With Advanced Melanoma (MK-3475-006/KEYNOTE-006)	YES	Melanoma	BIOLOGICAL: Pembrolizumab | BIOLOGICAL: Ipilimumab	ALL	ADULT, OLDER_ADULT	PHASE 3	834	INTERVENTIONAL	28/08/2013	03/03/2015	03/06/2019
Nivolumab	Study of BMS-936558 (Nivolumab) Compared to Docetaxel in Previously Treated Advanced or Metastatic Squamous Cell Non-Small Cell Lung Cancer (NSCLC) (CheckMate 017)	YES	Squamous Cell Non-Small Cell Lung Cancer	BIOLOGICAL: Nivolumab | DRUG: Docetaxel	ALL	ADULT, OLDER_ADULT	PHASE 3	272	INTERVENTIONAL	16/10/2012	17/11/2014	16/08/2021
Ipilimumab	Dacarbazine and Ipilimumab vs. Dacarbazine With Placebo in Untreated Unresectable Stage III or IV Melanoma	YES	Melanoma	DRUG: Ipilimumab | DRUG: Placebo | DRUG: Dacarbazine	ALL	ADULT, OLDER_ADULT	PHASE 3	681	INTERVENTIONAL	2006/08	2011/01	2013/10
